# Structural Characterization, DFT Calculation, NCI, Scan-Rate Analysis and Antifungal Activity against *Botrytis cinerea* of (*E*)-2-{[(2-Aminopyridin-2-yl)imino]-methyl}-4,6-di-*tert*-butylphenol (Pyridine Schiff Base)

**DOI:** 10.3390/molecules25122741

**Published:** 2020-06-13

**Authors:** Alexander Carreño, Dayán Páez-Hernández, Plinio Cantero-López, César Zúñiga, Jan Nevermann, Angélica Ramírez-Osorio, Manuel Gacitúa, Poldie Oyarzún, Felipe Sáez-Cortez, Rubén Polanco, Carolina Otero, Juan A. Fuentes

**Affiliations:** 1 Center of Applied Nanosciences (CANS), Facultad de Ciencias Exactas, Universidad Andres Bello, República 330, Santiago 8370186, Chile; dayan.paez@unab.cl (D.P.-H.); pliniocantero@gmail.com (P.C.-L.); 2 FONDECYT de Inicio, Facultad de Ciencias Exactas, Universidad Andres Bello, República 330, Santiago 8370186, Chile; angelicamosorio@gmail.com; 3 Instituto de Ciencias Naturales, Facultad de Medicina Veterinaria y Agronomía, Universidad de Las Américas, Sede Providencia, Manuel Montt 948, Santiago 7500972, Chile; cesar.zuniga.c@gmail.com; 4 Facultad de Ciencias de la Salud, Universidad Central de Chile, Lord Cochrane 417, Santiago 8330507, Chile; 5 Laboratorio de Genética y Patogénesis Bacteriana, Facultad de Ciencias de la Vida, Universidad Andres Bello, República 330, Santiago 8370186, Chile; jnevermannschell@gmail.com; 6 Facultad de Química y Biología, USACH, Av. L.B. O’Higgins 3363, Santiago 7254758, Chile; manuelgacitua@gmail.com; 7 Laboratorio de Análisis de Sólido (LAS), Facultad de Ingeniería y Facultad de Ciencias Exactas, Universidad Andrés Bello, República 330, Santiago 8370186, Chile; poldie.oyarzun@unab.cl; 8 Centro de Biotecnología Vegetal (CBV), Laboratorio de Fitopatógenos Fúngicos, Facultad de Ciencias de la Vida, Universidad Andres Bello, República 330, Santiago 8370186, Chile; fs.cortez92@gmail.com (F.S.-C.); rpolanco@unab.cl (R.P.); 9 Escuela de Química y Farmacia, Facultad de Medicina, Universidad Andres Bello, República 252, Santiago 8320000, Chile; maria.otero@unab.cl

**Keywords:** Pyridine Schiff base, intramolecular hydrogen bond, *Botrytis cinerea*

## Abstract

*Botrytis cinerea* is a ubiquitous necrotrophic filamentous fungal phytopathogen that lacks host specificity and can affect more than 1000 different plant species. In this work, we explored **L1** [(*E*)-2-{[(2-aminopyridin-2-yl)imino]-methyl}-4,6-di-*tert*-butylphenol], a pyridine Schiff base harboring an intramolecular bond (IHB), regarding their antifungal activity against *Botrytis cinerea*. Moreover, we present a full characterization of the **L1** by NMR and powder diffraction, as well as UV–vis, in the presence of previously untested different organic solvents. Complementary time-dependent density functional theory (TD-DFT) calculations were performed, and the noncovalent interaction (NCI) index was determined. Moreover, we obtained a scan-rate study on cyclic voltammetry of **L1**. Finally, we tested the antifungal activity of **L1** against two strains of *Botrytis cinerea* (B05.10, a standard laboratory strain; and A1, a wild type strains isolated from Chilean blueberries). We found that **L1** acts as an efficient antifungal agent against *Botrytis cinerea* at 26 °C, even better than the commercial antifungal agent fenhexamid. Although the antifungal activity was also observed at 4 °C, the effect was less pronounced. These results show the high versatility of this kind of pyridine Schiff bases in biological applications.

## 1. Introduction

Schiff base compounds are organic compounds containing an azomethine C=N group [1,2,3,4]. Pyridine Schiff bases present a full versatility, generally due to the substituents around the azomethine group, allowing their application in diverse areas, including the generation of antimicrobial and antifungal compounds [5,6]. Recently, pyridine Schiff bases constituted by a pyridine ring and a phenolic ring connected by an azomethine group were reported. The presence of a pyridine contributes to the antifungal activity in this kind of Schiff bases [7,8,9]. At present, the most used antifungal agents include triazoles, which exhibit good antifungal activity and a broad spectrum of action [10,11]. Triazoles, and some other nitrogen-containing heteroaromatic compounds (e.g., pyridine), have been reported to inhibit CYP51 class of cytochrome P450 enzymes by direct coordination of nitrogen with the heme iron (type II ligands) [7,12,13]. CYP51 inhibition, in turn, leads to inhibition of ergosterol biosynthesis in fungi with the concomitant accumulation of toxic metabolites and cell death [14,15,16]. In this sense, pyridine Schiff bases have great potential as antifungal agents, as previously described [7,8,9].

*Botrytis cinerea* is a ubiquitous necrotrophic filamentous fungal phytopathogen, virtually lacking host specificity, able to infect more than 1000 different plant species [17]. This plant pathogen can infect a wide range of leafy vegetable crops, fruits and flowers, including commercial plants, generating enormous damage, even in the post-harvest stage of the final products (rotting away the infected fruit) [18,19].

*Botrytis cinerea* can colonize plants and fruits during harvest, through vasculature tissue, pedicel removal, or surface fruit wounds due to inadequate manipulation [20]. Gray mold symptoms typically appear after 3 to 4 weeks of cold storage (4 °C), indicating that this period can be considered as a critical moment to prevent *Botrytis cinerea* dissemination [20,21].

**Figure 1:**
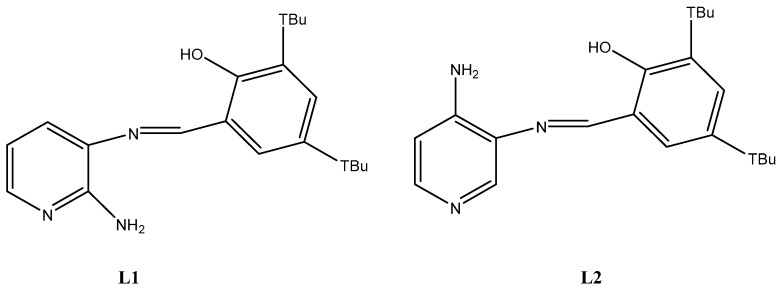
Chemical structure of (*E*)-2-{[(2-aminopyridin-2-yl)imino]-methyl}-4,6-di-*tert*-butyl-phenol (**L1**) and ((*E*)-2-{[(3-aminopyridin-4-yl)imino]-methyl}-4,6-di-*tert*-butyl-phenol) (**L2**).

In this work, we explored **L1** [(*E*)-2-{[(2-aminopyridin-2-yl)imino]-methyl}-4,6-di-*tert*-butylphenol] (Figure 1), a pyridine Schiff base harboring an intramolecular bond (IHB), regarding their antifungal effect against *Botrytis cinerea*. **L1** was previously described by our group, although its bioactivity against *Botrytis cinerea* was not assessed [9]. In addition, we present a full characterization of the **L1** structure by NMR and powder diffraction, also useful to corroborate the compound purity, as well as UV–vis, in the presence of previously untested different organic solvents. To complement these assays, we also performed time-dependent density functional theory (TD-DFT) calculations to corroborate the experimental UV–vis data, as well as the noncovalent interaction (NCI) index of **L1**, in order to better understand the nature of the IHB. Moreover, we performed a scan-rate study on cyclic voltammetry of **L1** to determine whether these processes are controlled by diffusion of species. Finally, we tested the antifungal activity of **L1** against two strains of *Botrytis cinerea* (B05.10, a standard laboratory strain; and A1, a wild type strains isolated from Chilean blueberries) [22], and other cell models including *Rhodotorula* spp. (yeast, unicellular fungus), *Klebsiella pneumoniae* (Gram-negative bacteria, human pathogen) and HeLa cells (human-derived cell line). We found that **L1** acts as an efficient antifungal agent only against *Botrytis cinerea*, and mainly at 26 °C, showing the versatility of this kind of pyridine Schiff bases in biological applications.

## 2. Results and Discussion

As stated above, **L1** synthesis and some characterizations have already been reported [4,9]. In this manuscript, we complement that characterization with additional data. **L1** is a yellow solid compound obtained with high yield. **L1** presented solubility both in apolar (e.g., chloroform) and polar solvents (e.g., acetonitrile, acetone and DMSO) at room temperature. Low solubility in either hexane or methanol was also observed, whereas **L1** was completely insoluble in water at room temperature. The FTIR spectrum (ATR), along with NMR assays (see below), were used to confirm the **L1** structure. In the FTIR spectrum, the range around 3500–3000 cm^−1^ presented bands that are generally considered as symmetric and asymmetric, due to νOH vibrations at 3469 cm^−1^. Two additional bands at 3255 and 3131 cm^−1^ were assigned to stretching vibrations mode (asymmetric and symmetric mode) of νNH (primary amino group) (See Figure S1 in the Supplementary Materials). These assignments are in agreement with previously reported studies [7,23]. It is important to remark that, due to the ATR limitations [24], the bands assigned to the –OH appeared to be thinner than bands obtained with the KBr pellet. Other authors reported broader bands in the range of 3500–3000 cm^−1^, due to stretching νOH group engaged in the IHB [23,25]. Nevertheless, we were unable to observe those bands in our FTIR, probably due to the overlapping with bands assigned to νNH. In our case, we assigned the bands at 2947 cm^−1^, 2905 cm^−1^ and 2863 cm^−1^ as νOH, νC–OH and νC–H, respectively. Other remarkable bands were observed at 1607 cm^−1^, and 1589 cm^−1^, assigned as νHC=N (azomethine), and νC=C, respectively. It is worth to underline that the azomethine group is usually assigned to around 1640 cm^−1^ [23,25]. The lower values observed for νHC=N can be explained by the interaction of the azomethine and the hydroxyl groups, both involved in the IHB, as previously reported for similar Schiff bases [23]. Furthermore, the frequency analysis for the optimized geometry obtained by DFT calculations (CAM-B3LYP) confirmed this assignment (data not shown).

Previously, the intramolecular hydrogen bond (IHB) present in **L1** was assessed, by ^1^H-NMR, under different organic deuterated solvents (CDCl_3_, acetone-_d6_ and DMSO-_d6_), showing the stability of this IHB [4,9]. In order to corroborate the stability of the IHB, in the present work, we assessed other previously untested solvents, including acetone-_d6_ (control), CD_2_Cl_2_ and methanol-_d4_ by ^1^H NMR, ^13^C-NMR, DEPT, HHCOSY at 25 °C (See Figures S2 to S16 in the Supplementary Materials, which include the aromatic expanded regions). It is essential to mention that the most important signals, i.e., the –OH and –NH_2_ groups, appeared at 13.29 ppm and 5.51 ppm in acetone-_d6_, respectively, and 12.94 ppm and 5.23 ppm in the case of CD_2_Cl_2_. Regarding the methanol-_d4_, these signals vanished due to the deuterated exchange, confirming the assignment.

We performed experiments using different solvents of different polarities to assess the stability of the IHB, due to possible interactions with the solvents. In **L1**, the IHB is formed between the azomethine and the hydroxyl group from the phenolic ring. In this context, in a less stable IHB, we expect a strong interaction of this IHB with the more polar solvents, such as methanol or DMSO, due to intermolecular interactions. These interactions would affect the assigned signals in ^1^H-NMR assays. By contrast, in a more stable IHB, we expect no changes, regardless of the solvent used. This strategy has been previously used to assess the stability of the IHB in other pyridine Schiff bases [8]. As stated, ^1^H-NMR showed the proton from the hydroxyl group involved in the IHB was assigned around 13 ppm in all the deuterated solvents tested (Figure S2 for acetone-_D6_, and Figure S7 for CD_2_Cl_2_ in the Supplementary Materials). If the hydroxyl group had presented an interaction with the solvent, affecting the IHB, we were observed a sharp shift in the position of this band due to a proton transfer. Conversely, the band remained in the same position (around 13 ppm). On the other hand, the proton corresponding to the azomethine group (–CH=N–) appeared around 8.88–8.56 ppm as a singlet (Figures S2, S7 and S12 in the Supplementary Materials), whereas the carbon corresponding to this same group appeared at 165 ppm (Figures S4, S9 and S14 in the Supplementary Materials) in all solvents tested.

These results corroborate the high stability of IHB and, more importantly, the purity of the **L1** powder that was subsequently used for biological tests. Since our aim in this work is to find out whether **L1** could be used as an antifungal agent against *Botrytis cinerea*, a fungus able to infect at storage conditions (i.e., at 4 °C) [20,21], we also tested the **L1** structure at that temperature. For that reason, we determined the ^1^HNMR spectrum of **L1** in CD_2_Cl_2_ at 4 °C. As shown in Figure S17 (See Supplementary Materials), **L1** presented a similar signal pattern to that found at 25 °C with the same solvent (Figure S7 in the Supplementary Materials), indicating that **L1** maintains its conformational structure both at 25 °C and 4 °C.

The absorption spectra of **L1** were studied in four organic solvents of increasing polarity (hexane, chloroform, methanol and DMSO) at room temperature. In the presence of either hexane or methanol, we observed that **L1** presented three intense absorption bands around 236 nm, 274 nm and 368 nm (Figure S18 in the Supplementary Materials). The band around 236 nm and 274 nm corresponded to the π → π* of the aromatic rings, by analogy with similar organic compounds [26,27]. The band around 368 nm was assigned to a combination of n → π* and π → π* ligand transitions [23,28]. The TD-DFT calculations were used to study the electronic structure and investigate the nature of these transitions. The band assignment to π → π* within the aromatic rings (pyridine and phenolic rings) in the Schiff base exhibited a composition of HOMO-2 → LUMO. On the other hand, the bands assigned to a combination of n → π* and π → π* transitions involved HOMO → LUMO compositions in all the solvents tested. This point will be discussed below. We observed that the band corresponding to the azomethine group (involved in the IHB) appeared at lower energy (around 368-377 nm with ε < 23.77 × 103 mol^−1^ dm^3^ cm^−1^) compared with the other bands corresponding to the pyridine and phenolic rings (Table S2 in the Supplementary Materials). This fact can be explained by the delocalized, resonating electron cloud involved in the formation of the IHB, as reported for other similar Schiff bases [4].

On the other hand, in chloroform, we observed two bands at 274 nm (π → π*) and 372 nm (n → π* and π → π*); by contrast, only one band was obtained with DMSO (378 nm; n → π* and π → π*) (Figure S18 and Table S2 in the Supplementary Materials). This result can be explained by the nature of the organic solvent used. In general, all solvents present a cut-off wavelength, where the DMSO cut-off wavelength is high (270 nm) [29], a fact corroborated by our results (Figure S18 in the Supplementary Materials). Nevertheless, the study of bands with higher wavelengths can be performed without any problem. In Figure S18 (Supplementary Materials), we observed that **L1** exhibited two groups of bands. One group around 236–277 nm, and another band around 370 nm. Although the bands around 236–277 nm are not visible with DMSO, due to its high cut-off, these bands were easily distinguished with the other solvents tested (i.e., hexane, methanol, chloroform). On the other hand, the DMSO was useful to observe the band around 370 nm, which involves the IHB.

In a protic solvent, the IHB may weaken in favor of a solute–solvent interaction. This balance of the increased internal energy and the stabilizing effect between the solute–solvent interactions could regulate new conformer composition in the liquid phase, such as tautomeric equilibrium (keto-enolic) [30]. This effect could be observed as modifications in the bands associated with the IHB in the UV–vis spectrum, including a band splitting or the presence of a shoulder, as previously described for other Schiff bases with an IHB [31,32]. In the case of **L1**, we were unable to observe a split or significant modifications in the band associated with the IHB (around 370 nm) in the UV–vis spectrum. In other words, we observed no significant shifts in the IHB band, independently on the solvent used (hexane, chloroform, methanol or DMSO). In **L1**, the formation of an intramolecular quasi-aromatic hydrogen bonding was reinforced through the delocalization of the π-electrons stabilizing the molecule and strengthening the hydrogen bond [23,26]. The NCI theoretical study confirmed this fact (see below).

**Figure 2:**
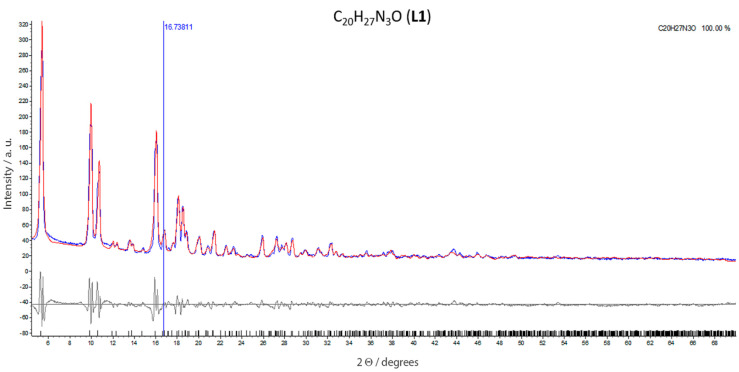
Pattern for **L1** [(*E*)-2-{[(2-aminopyridin-2-yl)imino]-methyl}-4,6—di-*tert*-butyl-phenol]: The observed (red line) and calculated (blue line), and difference X-ray powder diffraction patterns (bottom), are shown. The difference pattern is plotted at the same scale as the other patterns up to 60° 2θ. At higher angles, the scale has been magnified five times. Red line: experimental pattern; blue line: calculated pattern; gray line: difference between the calculated and experimental pattern.

In order to corroborate the absence of structural isomers and to check possible geometrical changes produced in the solid state, we further characterized **L1** with X-ray powder diffraction. Since the **L1** powder was the source of subsequent tests shown in this work, this characterization is crucial. To perform X-ray powder diffraction, **L1** was mounted on a zero-background cell, and the pattern was determined in a Bruker D8 Advance diffractometer equipped with a LynxEye position-sensitive detector [33]. Using the corrected diffraction data of **L1** (C_20_H_27_N_3_O, M = 325.45 g/mol), accurate unit-cell parameters were obtained with a = 17.0520 Å, b = 10.6445 Å, c = 10.5946 Å, *β* = 102.12°, *V* =1880.1 (Å^3^) and density 1.15 g cm^−3^. The experimental XRD pattern of **L1** is shown in Figure 2. All lines were successfully indexed using the Pawley method into the TOPAS 4.2 program. By comparing powder X-ray diffraction data with single crystal data (previously published) [34], we found the same structure type with space group P21/c (N° 14), corroborating the purity of **L1**. Table 1 provides the X-ray data of **L1**, including cell parameters, Z values and calculated density, and refinement results, along with crystallographic data (in parenthesis) for comparison. Abnormal radiations were reduced using polynomial background (third order). The Fourier polynomials, as well as the fluorescence emission, were reduced using a Silicon sample holder for a correct refinement of powder sample, using radiation CuKa [35,36,37,38,39].

These results corroborated the crystallographic structure of **L1**, showing that no geometrical changes occur in the solid state (powder). In addition, we ruled out the presence of structural isomers, and corroborate the **L1** purity. This powder was used for the next experiments reported in this manuscript, including biological tests.

### 2.1. Electrochemical Studies

**Figure 3:**
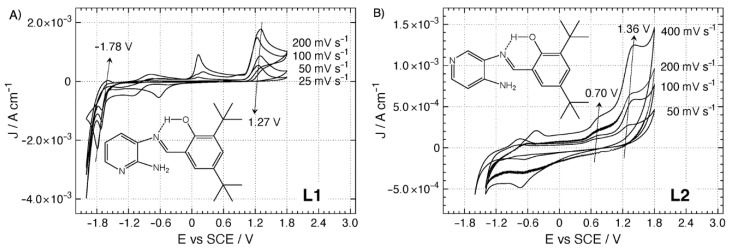
Scan-rate study for **L1** and **L2**. Interphase: Pt|1.0 10^−2^ M of compound +1.0 × 10^−1^ M TBAPF_6_ in anhydrous CH_3_CN.

Previously, the electrochemical profile of **L1** was reported, showing that exhibited only one irreversible oxidation process at +1.27 V while the process near +0.20 V was discarded since it was only visible when the voltammogram reaches the most negative limit [4]. On the other hand, **L2** showed two irreversible oxidation peaks at +0.70 and +1.36, respectively [9]. To further extend the electrochemical studies of **L1**, and compare its profile with the **L2** profile, we performed a scan-rate study on cyclic voltammetry responses to check whether reported redox processes by Carreño et al. [4,9] are controlled by diffusion of species or not [40,41]. The scan-rate study is presented in Figure 3. As reported [4,42], **L1** possesses an irreversible oxidation at 1.27 and a reversible reduction at −1.78 V, while **L2** displays two irreversible oxidations at 0.70 and 1.36 V vs. SCE, respectively (see Table S3 in the Supplementary Materials). The scan-rate analysis considered a comparison between graphics of Current density peaks vs. Scan Rate and Current density peaks vs. (Scan Rate)^1/2^, indicating that electrochemical processes for **L1** are likely controlled by diffusion; by contrast, those for **L2** are controlled by other phenomena. Moreover, after analysis of graphics constructed with Log(Current-density peaks) vs. Log(Scan Rate) (see Figures S19 and S20), it was concluded that **L1**-electrochemical processes are controlled by mass-transfer (diffusion) while those found for **L2** are controlled by adsorption [43,44] (see Tables S4 and S5 in the Supplementary Materials).

It has been determined that regular pyridines can be strongly adsorbed over polycrystalline platinum electrodes [45]. Regarding **L1** or **L2**, it is more feasible to imagine that situation for **L2** since the free non-bonding electron pair from the nitrogen at the pyridine ring shows less steric hindrance from neighboring moieties and can thus more easily coordinate with Pt atoms over the platinum working electrode. This phenomenon could also contribute to explain the electrochemical differences between **L1** and **L2**.

### 2.2. DFT Calculations

**L1** geometry was obtained from the B3LYP hybrid exchange/correlation (XC) functional and Gaussian basis set 6-311+G (2d,p) [46,47]. The geometrical parameters agree with the X-ray crystallographic data [34]. Time-dependent density functional theory (TD-DFT) calculations were used to find the energies for the first 100 excitations [48,49] and elucidate the UV–vis transitions in four different solvents. The UV–vis calculated spectra in hexane, chloroform, methanol and DMSO showed absorption bands centered approximately at 280 nm and 395 nm (Table S6 in the Supplementary Materials) for the **L1** compound, which were assigned to π → π* and a combination of n → π* and π → π* transitions, respectively. The calculated transitions showed that excitations were mainly associated with π → π* in the Schiff base and exhibited a composition of HOMO-2 → LUMO (88%) in hexane, HOMO-2 → LUMO (92%) in chloroform, HOMO-2 → LUMO (94%) in methanol and HOMO-2 → LUMO (93%) in DMSO. Under this framework, the assigned bands as π → π* and n → π* transitions involved HOMO → LUMO compositions in all the solvents studied. The comparison between the different solvents showed no significant shift in the computed UV–vis spectra when the polarity changed, confirming the stability of the intramolecular hydrogen bond, in agreement with the experimental data (Figures S18 and S21 in the Supplementary Materials). On the other hand, the molecular-orbital diagram of **L1**, the HOMO is located at the phenolic ring and the azomethine group (–C=N–), whereas LUMO is distributed along the phenolic and pyridine ring. The HOMO-2 is found in the phenolic and aromatic rings. A qualitatively molecular orbital diagram is shown in Figures S22, S23 and S24 in the Supplementary Materials.

Finally, TD-DFT calculations were performed using the standard CAM-B3LYP/TZ2P level of theory. Solvation effects, simulated by the conductor-like screening model (COSMO) [50,51]. COSMO (“COnductor-like Screening MOdel”), is a calculation method for determining the electrostatic interaction of a molecule with a solvent. COSMO treats each solvent as a continuum with a permittivity ε and therefore belongs to the “continuum solvation” group of models. In this context, COSMO is only an approximation that does not necessarily reflect the solute-solvent interaction in the liquid state. For that reason, the DMSO wavelength cut-off was not represented in the computed spectrum (Figure S21 in the Supplementary Materials).

### 2.3. Noncovalent Interaction Index (NCI)

Noncovalent interactions index studies provide an understanding of many chemical and biological processes [52,53,54]. The algorithms for mapping and analyzing the noncovalent interactions had been derived from the electronic and kinetic-energy densities [55,56,57]. In this sense, the NCI index identifies interactions in a chemical system solely based on the electron density, and its derivative was given by Equation (1). A graphical index in 2D provides a characterization of this interaction [58]. To further characterize the IHB in **L1**, and its stability, the first step in the decoupling process of forces defining noncovalent interactions involved the inclusion of the IHB position in the real space. Our NCI analysis predicted that the nitrogen lone pair promotes the *E*-conformation in the correct orientation to participate in an n → π* interaction. The reduced gradient density at low-density regions verifies the presence of noncovalent interactions, where each point in this region is correlated with the second eigenvalue of the electron density Hessian (λ) times. The importance of the λ values accounts the type of binding force: negative λ values (λ < 0) indicate attractive forces, such as hydrogen bonds; while weak interactions or repulsive forces are inferred by λ = 0 and λ > 0, respectively.

**Figure 4:**
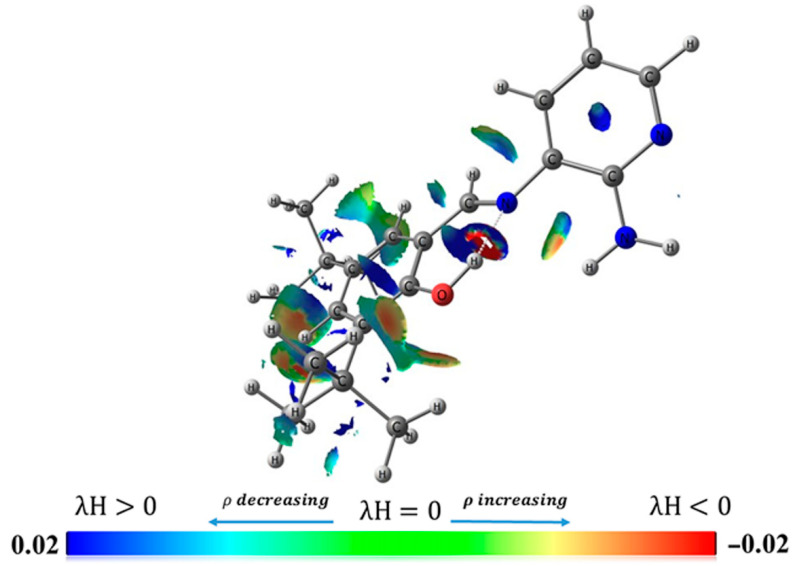
Noncovalent interaction (NCI) analysis of **L1**. Bottom: the NCI plot gradient isosurface (0.6 a.u.) for five local and global minima structures (kcal/mol^−1^). The NCI color scale is −0.02 < λH > 0.02 a.u. These calculations were performed at the B3LYP/6-31G** level.

Figure 4 corroborates the presence of a strong intramolecular hydrogen bond (λH < 0) in **L1** between the hydroxyl group and nitrogen atom in the azomethine group. The NCI plot gradient isosurface (0.6 a.u.) for five local and global minima structures (A = 0.000, B = 0.100, C = 1.734, D = 2.183 and E = 3.183, all units are in kcal/mol^−1^) is shown. The isosurfaces color scale is a blue-green-red scale according to the strength and type of interaction (attractive or repulsive), similar to that previously reported [59,60,61]. The red color indicates a strong, attractive interaction; the green color indicates a weak Van der Waal interaction, and the blue color shows a strong non-bonded overlap. The presence of the strong IHB was also supported by the ^1^H-NMR analysis and UV–vis in different organic solvents (see above).

Thus, our results corroborate the presence of the IHB in **L1**, indicating that this structure is present both in the solid and liquid state. This property is particularly relevant in the design of stable antifungal agents.

### 2.4. Cytotoxicity Assays

#### **L1** Presents Antifungal Activity against *Botrytis cinerea*

As stated above, *Botrytis cinerea* is a fungal phytopathogen, causing the “grey mold” disease in a wide range of plants, including species of importance in the food industry [18]. Previously, we observe the antifungal activity of **L1** against *Cryptococcus* spp. (yeast), although **L1** seemed to be ineffective against *Candida albicans* [9]. In this context, we explored the potential fungicidal activity of **L1** against *Botrytis cinerea*. Thus, we first tested the **L1** antifungal effect at 26 °C against *Botrytis cinerea* B05.10, a standard laboratory strain [22], and *Botrytis cinerea* A1, a faster-growing strain isolated from blueberries in the field, in Chile [62].

**Figure 5:**
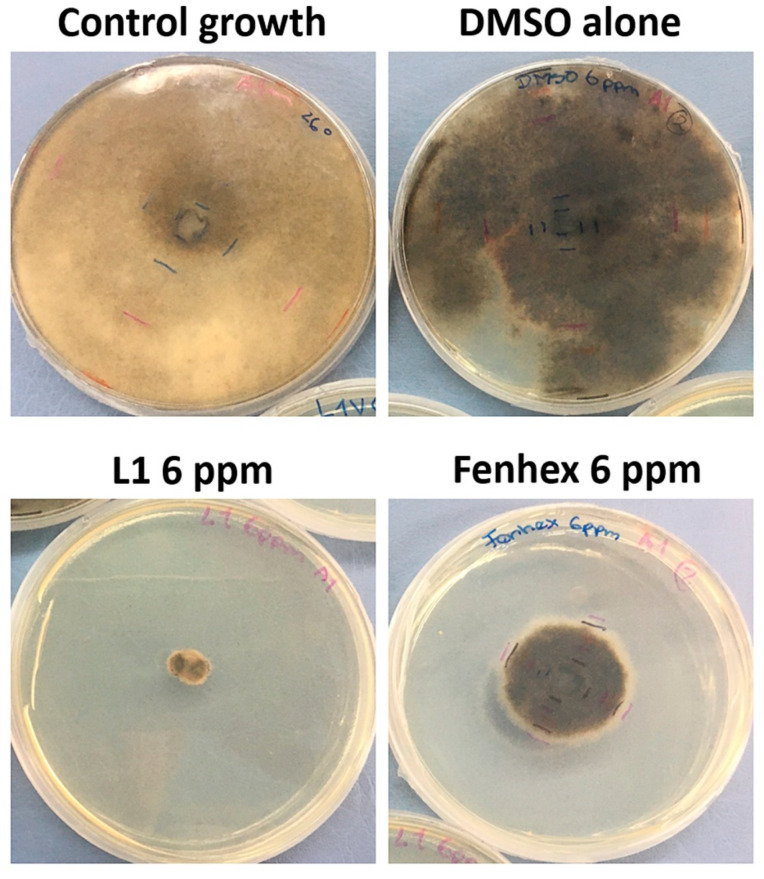
Antifungal effect against *Botrytis cinerea* A1 (at 26 °C) exerted by **L1** in a dose-dependent manner (12 days of incubation). Inhibition of mycelial growth (by measuring the colony diameter) was observed in the presence of **L1** (6 ppm) and compared with the commercial fungicide fenhexamid (6 ppm). Since both **L1** and fenhexamid stock were dissolved in DMSO (vehicle), DMSO alone was also tested, adding the same concentration used with either **L1** or fenhexamid in each case (0.045% *v/v*). The figure shows a representative experiment, showing the fungal colony growth.

**Figure 6:**
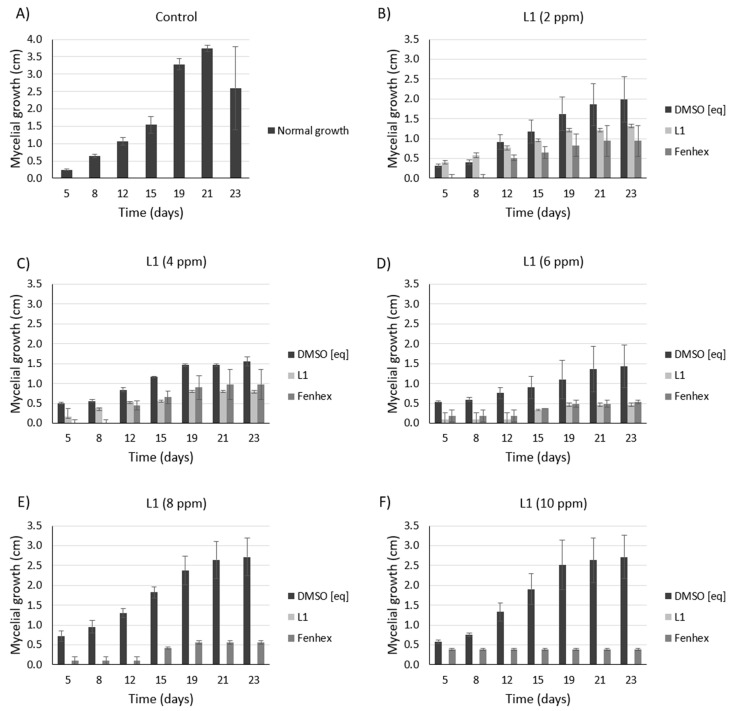
Antifungal effect against *Botrytis cinerea* B05.10 (at 26 °C) exerted by **L1** in a dose-dependent manner. Inhibition of mycelial growth (by measuring the colony diameter) was observed in the presence of **L1** ((**A**): 0 ppm, (**B**): 2 ppm, (**C**): 4 ppm, (**D**): 6 ppm, (**E**): 8 ppm, (**F**): 10 ppm) and compared with the commercial fungicide fenhexamid ((**A**): 0 ppm, (**B**): 2 ppm, (**C**): 4 ppm, (**D**): 6 ppm, (**E**): 8 ppm, (**F**): 10 ppm). Since both **L1** and fenhexamid stock were dissolved in DMSO (vehicle), DMSO alone was also tested, adding the same concentration used with either **L1** or fenhexamid in each case ((**A**): 0% *v/v*, (**B**): 0.015% *v/v*, (**C**): 0.030% *v/v*, (**D**): 0.045% *v/v*, (**E**): 0.060% *v/v*, (**F**): 0.075% *v/v*) (DMSO [eq]). In all cases, the experiments were performed in biological triplicate.

**Figure 7:**
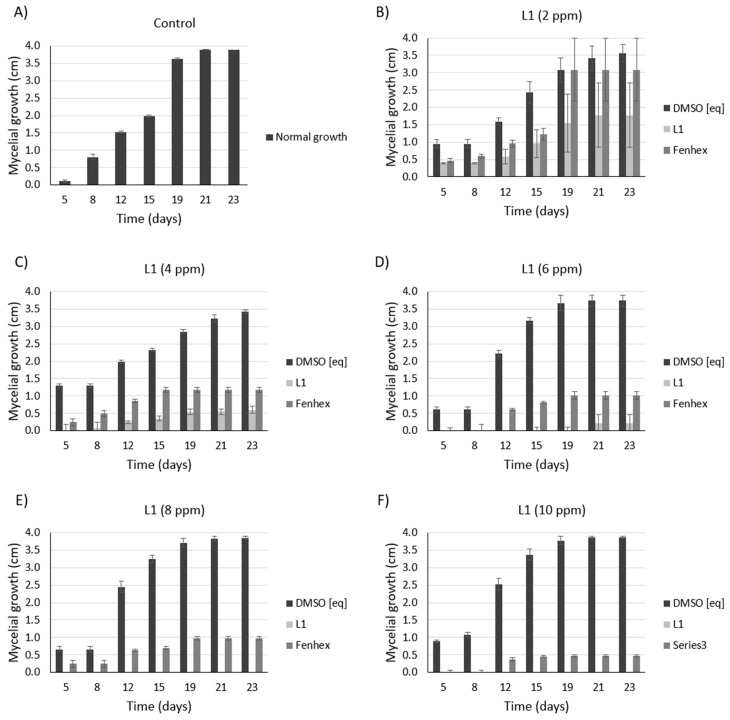
Antifungal effect against *Botrytis cinerea* A1 (at 26 °C) exerted by **L1** in a dose-dependent manner. Inhibition of mycelial growth (by measuring the colony diameter) was observed in the presence of **L1** ((**A**): 0 ppm, (**B**): 2 ppm, (**C**): 4 ppm, (**D**): 6 ppm, (**E**): 8 ppm, (**F**): 10 ppm) and compared with the commercial fungicide fenhexamid ((**A**): 0 ppm, (**B**): 2 ppm, (**C**): 4 ppm, (**D**): 6 ppm, (**E**): 8 ppm, (**F**): 10 ppm). Since both **L1** and fenhexamid stock were dissolved in DMSO (vehicle), DMSO alone was also tested, adding the same concentration used with either **L1** or fenhexamid in each case ((**A**): 0% *v/v*, (**B**): 0.015% *v/v*, (**C**): 0.030% *v/v*, (**D**): 0.045% *v/v*, (**E**): 0.060% *v/v*, (**F**): 0.075% *v/v*) (DMSO [eq]). In all cases, the experiments were performed in biological triplicate.

As shown in Figure 5, Figure 6 and Figure 7, **L1** exerted a dose-dependent antifungal activity at 26 °C, as observed by the inhibition of mycelial growth diameter. As a control, we tested the solvent alone (DMSO, vehicle), which exerted a detrimental effect only on the *Botrytis cinerea* B05.1 strain (Figure 6 and Figure 7), whereas DMSO hardly affected the growth of *Botrytis cinerea* A1 strain (Figure 7). This result corroborates the antifungal effect of **L1**, which is slightly better than that obtained with the commercial fungicide fenhexamid. Interestingly, this difference is more evident with the *Botrytis cinerea* A1 strain (compare Figure 6 with Figure 7).

**Figure 8:**
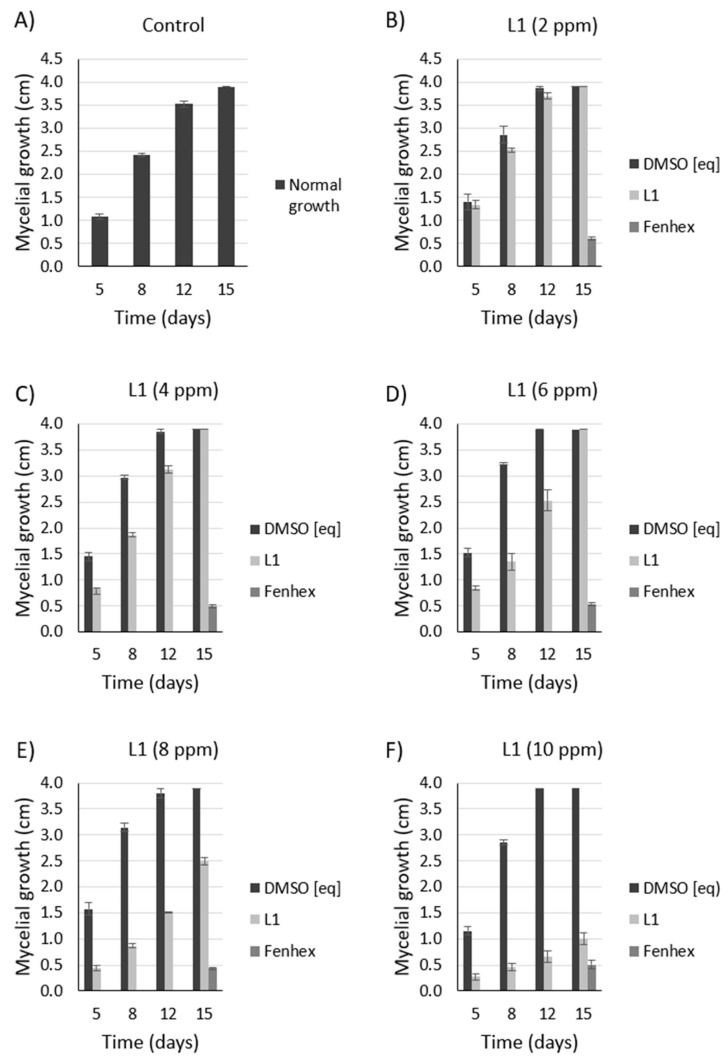
Dose-dependent antifungal effect of **L1** against *Botrytis cinerea* B05.10 at 4 °C. Inhibition of mycelial growth (by measuring the colony diameter) was observed in the presence of **L1** ((**A**): 0 ppm, (**B**): 2 ppm, (**C**): 4 ppm, (**D**): 6 ppm, (**E**): 8 ppm, (**F**): 10 ppm) and compared with the commercial fungicide fenhexamid ((**A**): 0 ppm, (**B**): 2 ppm, (**C**): 4 ppm, (**D**): 6 ppm, (**E**): 8 ppm, (**F**): 10 ppm). Since both **L1** and fenhexamid stock were dissolved in DMSO (vehicle), DMSO alone was also tested, adding the same concentration used with either **L1** or fenhexamid in each case ((**A**): 0% *v/v*, (**B**): 0.015% *v/v*, (**C**): 0.030% *v/v*, (**D**): 0.045% *v/v*, (**E**): 0.060% *v/v*, (**F**): 0.075% *v/v*) (DMSO [eq]). In all cases, the experiments were performed in biological triplicate.

**Figure 9:**
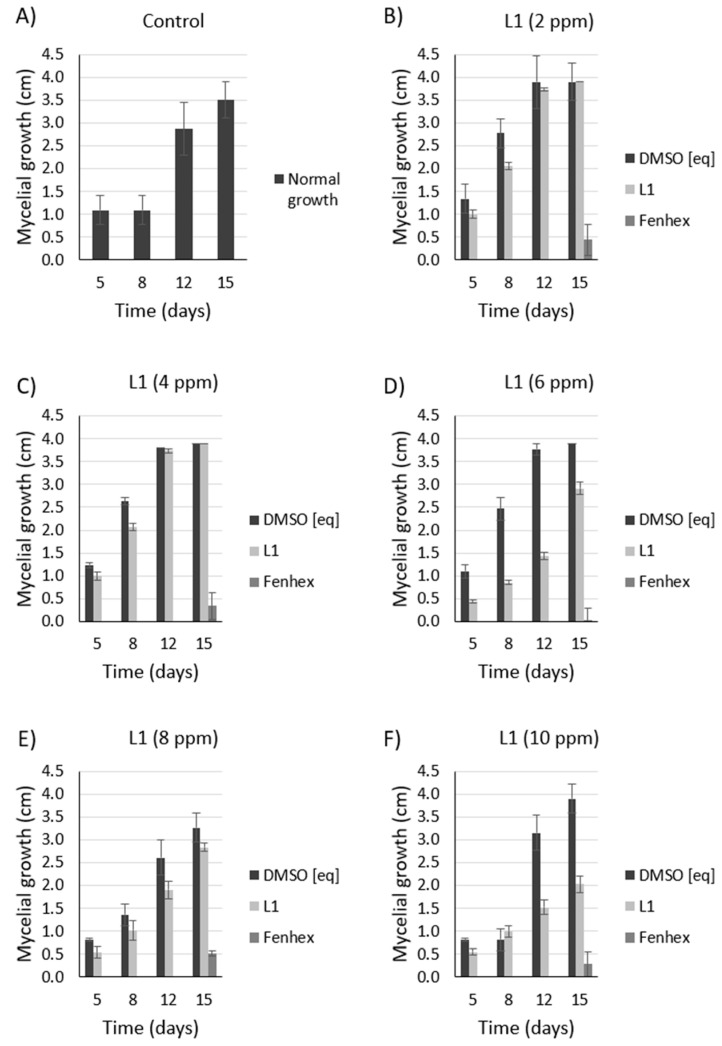
Dose-dependent antifungal effect of **L1** against *Botrytis cinerea* A1 at 4 °C. Inhibition of mycelial growth (by measuring the colony diameter) was observed in the presence of **L1** ((**A**): 0 ppm, (**B**): 2 ppm, (**C**): 4 ppm, (**D**): 6 ppm, (**E**): 8 ppm, (**F**): 10 ppm) and compared with the commercial fungicide fenhexamid ((**A**): 0 ppm, (**B**): 2 ppm, (**C**): 4 ppm, (**D**): 6 ppm, (**E**): 8 ppm, (**F**): 10 ppm). Since both **L1** and fenhexamid stock were dissolved in DMSO (vehicle), DMSO alone was also tested, adding the same concentration used with either **L1** or fenhexamid in each case ((**A**): 0% *v/v*, (**B**): 0.015% *v/v*, (**C**): 0.030% *v/v*, (**D**): 0.045% *v/v*, (**E**): 0.060% *v/v*, (**F**): 0.075% *v/v*) (DMSO [eq]). In all cases, the experiments were performed in biological triplicate.

Since symptoms of the grey mold disease are usually evident even at cold temperatures (4 °C), a normal condition for fruits and vegetables that must be transported or stored, we tested the antifungal effect of **L1** at 4 °C. We observed that **L1** exerted a dose-dependent antifungal activity against *Botrytis cinerea* B05.1 and A1 (Figure 8 and Figure 9), although, in this case, the antifungal effect is weaker compared to that obtained at 26 °C. At 4 °C, the fenhexamid was more efficient than **L1**.

Previous comparative studies have shown that **L2** (((*E*)-2-{[(3-aminopyridin-4-yl)imino]-methyl}-4,6-di-*tert*-butyl-phenol), Figure 1), and not **L1**, exerted a cytotoxic effect against yeasts (*Cryptococcus* spp. MIC: 4.468 ppm) [9]. It has been reported that some nitrogen-containing aromatic compounds, such as pyridine Schiff bases, might present antifungal properties [7,13]. Evidence has been presented suggesting that the availability of the nitrogen in the pyridine ring could affect the antifungal property since some pyridine Schiff bases exhibit weak or null antifungal effect [7,9]. When **L1** and **L2** structures are compared, we can observe that the nitrogen in the pyridine ring is more available in **L2** than in **L1**, providing a possible explanation for the better antifungal effect obtained with **L2** against yeasts in comparison with **L1** [42]. In general, it has been proposed that the nitrogen in the pyridine ring might be necessary for the antifungal activity of Schiff bases, remarking the structure-bioactivity relationship [7,8,63]. Nevertheless, in this work, we found that **L1** exhibits a potent antifungal effect against *Botrytis cinerea* at 26 °C, even better than the commercial antifungal agent fenhexamid. In addition, although **L1** was less potent at 4 °C, we found similar results to those reported for **L2** under similar conditions [42], suggesting that antifungal effect of this kind of Schiff bases against filamentous fungi such as *Botrytis cinerea* cannot be freely extrapolated from results obtained with yeast without an experimental approach. On the other hand, **L1** was more cytotoxic at 26 °C than at 4 °C (see above). Since the **L1** structure remained unaltered in both 26 °C and 4 °C (Figure S17), possibly the decreased cytotoxicity at 4 °C may be attributed to changes in the *Botrytis cinerea* metabolism in cold environments, as inferred by the modulation of the fungal transcriptome under such condition [64].

Finally, in order to assess whether **L1** can exert a cytotoxic effect against other cell models, including a clinical isolate of *Rhodotorula* spp. (yeast, unicellular fungus) *Klebsiella pneumoniae* (Gram-negative enterobacteria) and the HeLa cells (epithelial cell line derived from cervical cancer cells). For *Rhodotorula* spp. and *K. pneumoniae*, we were unable to see a cytotoxic effect of **L1** (Table S7 in the Supplementary Materials), similar to that previously described for *Candida albicans* (yeast, unicellular fungus) and *Salmonella enterica* (Gram-negative enterobacteria), respectively [9]. In the case of HeLa cells, we found that **L1** (or **L2**) exerted an indistinguishable effect from the vehicle alone, showing low or almost null cytotoxic effects against these cells, even at 200 ppm (100-fold the amount needed to affect the *B. cinerea* negatively) (Figure S25 in the Supplementary Materials). This result suggests that **L1** could exert a differential effect primarily against *Botrytis cinerea*, and especially at 26 °C, although more experimentation is needed to determine whether **L1** is innocuous for human cells.

## 3. Material and Methods

### 3.1. Instrumentation

FTIR spectra were obtained on a Bruker Vector-22 FTIR spectrophotometer, ATR UV–vis spectra were performed using a UV–vis-NIR scanning spectrophotometer Perkin Elmer Model Lamda 35 in hexane, chloroform, methanol and DMSO as solvents at room temperature. ^1^H-NMR, ^13^C-NMR, HHCOSY and DEPT spectra for **L1** were recorded on a Bruker AVANCE 400 spectrometer at 400 MHz at 25 °C. HHCOSY (two-dimensional nuclear magnetic resonance spectroscopy, 2D NMR) is a set of nuclear magnetic resonance spectroscopy (NMR) methods which give data plotted in a space defined by two frequency axes rather than one, providing more information about a molecule than one-dimensional NMR spectra and especially useful in determining the structure of a molecule. DEPT (Distortionless Enhancement by Polarization Transfer) is a double resonance pulse program that transfers polarization from an excited nucleus to another. This is a useful a tool for 13C peak assignments. Samples were dissolved in some common deuterated organic solvents according to increasing polarity (dichloromethane-_d2_ [CD_2_Cl_2_], acetone-_d6_ and methanol-_d4_), using tetramethylsilane as an internal reference. When indicated, ^1^H-NMR of **L1** was measured at 4 °C in dichloromethane-_d2_ (CD_2_Cl_2_).

For UV–vis spectra, the stock dilution of **L1** (1.25 × 10^−3^ mol/L) was performed using methanol as the solvent in a glass flask. The calibration curve (5 points) in each case was prepared in the same measuring cell, adding an aliquot of 20 µL, 40 µL, 60 µL, 80 µL or 100 µL of the stock solution to 3000 µL of the tested solvent in each case (hexane, chloroform, methanol and DMSO), with a final concentration of 8.278 × 10^−6^ M, 1.645 × 10^−5^ M, 2.451 × 10^−5^ M, 3.247 × 10^−5^ M, 4.032 × 10^−5^ M, respectively. Measures were performed in a 1-cm quartz cuvette.

For power diffraction analysis (Bruker D8 Advance diffractometer), the **L1** powder was obtained by grinding the compounds. It is important to underline that this same powder was used for biological assays.

### 3.2. Electrochemical Measurements

For complementary electrochemical experiments, the working solution contained 0.01 mol/L of the respective compound with 0.1 mol/L tetrabutylammonium hexafluorophosphate (TBAPF_6_, supporting electrolyte) in CH_3_CN. Before each experiment, the working solution was purged with high purity argon, and an argon atmosphere was maintained during the whole experiment. A polycrystalline non-annealed platinum disc (2 mm diameter) was used as the working electrode. A platinum gauze of a large geometrical area, separated from the main cell compartment by a fine sintered glass, was used as the counter electrode [65,66]. All potentials quoted in this paper are referred to as an Ag/AgCl electrode in tetramethylammonium chloride to match the potential of a saturated calomel electrode (SCE), at room temperature. All electrochemical experiments were performed at room temperature on a CHI900B bipotentiostat interfaced to a PC running the CHI 9.12 software that allowed experimental control and data acquisition, as previously reported for other determinations [67,68].

### 3.3. Quantum Chemistry

Theoretical computations were performed using density functional theory (DFT) with the B3LYP hybrid exchange/correlation (XC) functional, which includes the non-local exchange term, with three parameters of Becke and the correlation term of Lee-Yang-Parr [69,70]. Gaussian basis set 6-311+G (2d,p) was used. Molecular geometry of the ground states was fully optimized, and the frequency analysis was performed after each geometry optimization, were obtained only positive frequencies verifying local minima. TD-DFT calculations were performed in four different organic solvents phase (see below) by using the standard CAM-B3LYP/TZ2P level of theory [50,71,72,73]. In this context, the hybrid exchange-correlation functional CAM-B3LYP combines the hybrid qualities of B3LYP, and the long-range correction previously reported [50]. Solvation effects were simulated by the conductor-like screening model (COSMO) [74,75,76,77] using hexane, chloroform, methanol and DMSO as solvents. To reveal possible noncovalent interactions, such as hydrogen bonds, steric repulsion, van der Waals interactions, noncovalent interaction index (NCI) was performed. NCI is based on the electron density and its derivatives, which enables the identification of noncovalent interactions on the reduced density gradient (S) at low-density regions (ρ) [52,78,79,80]. This analysis provides a graphical index (2D plot), which allows the characterization of the interactions mentioned before. In this framework, the reduced density gradient is given by Equation (1):

(1) $$S=\frac{1}{2{\left(3\pi^{2}\right)}^{1/3}}\frac{\nabla \rho }{\rho^{4/3}}$$

When a weak inter- or intramolecular interaction is present, there is a crucial change in the reduced gradient between the interacting atoms, producing critical density points between interacting fragments, associated with each critical point. Since the behavior of s at low densities is dominated by ρ, s tends to diverge, except in regions around a density critical point, where ρ dominates, and s approaches zero [58,81,82].

### 3.4. X-Ray Rietveld Refinements and Powder Reference Patterns

**L1** was mounted on a zero-background cell. The X-ray powder patterns were measured on a Bruker D8 Advance diffractometer (40 kV, 30 mA, 5°–70° 2θ in 0.020 steps, 0.5 s step−1) equipped with a LynxEye position-sensitive detector. The lower window of the detector electronics was increased using Si-Einkristalle at the default value of 0.11–0.19 V to minimize the effects of fluorescence. The Rietveld refinement technique [83,84,85] with software suite Diffrac TOPAS 4.2 [33,86,87,88] was used to determine the **L1** structure. The single-crystal structure of **L1**, used to compare our results, has been previously reported [34]. The parameter fundamentals (profile function #1) were used for the refinements [4,89]. Background function #0 (shifted Chebyshev function with 3 terms) in combination with the CW X-ray profile (functions #1) were used for the refinements [33,90,91,92]. (Breger et al., 2005; Hunger et al., 1999; Liu et al., 2011; Salvi et al., 2015). Profile of cell coefficient parameters, peak shift, sample convolutions and preferred orientation was refined. Peak tails were ignored where the intensity was below 0.005 times the peak. Reference patterns were obtained with a Rietveld pattern decomposition technique. Using this technique, reported peak positions were derived from the extracted integrated intensities and positions calculated from the lattice parameters. When peaks were not resolved with the resolution function, intensities were summed and an intensity-weighted d-spacing was reported. Table 1 gives the pertinent atomic coordinates and displacement parameters. **L1** is monoclinic with space group *P21/c* (No 14), *Z* = 4. Additionally, we reported the single crystal data of **L1** in parenthesis in order to underline that these results were in agreement with previous reports [34]. Positional (x, y, z) and displacement parameters for **L1** is shown in Table S1 in the Supplementary Materials.

### 3.5. Material

All starting materials (reactants and solvents PA grade) were purchased from Merck and Aldrich and used with no further purification. Acetonitrile (CH_3_CN for HPLC grade) was drying molecular sieves and purged under argon gas for electrochemical applications.

### 3.6. Antimicrobial activity

*Botrytis cinerea* strain B05.10 (a standard laboratory strain) [22] and A1 (a wild type strain isolated from Chilean blueberries) were obtained from the Phytopathogenic Fungi Laboratory at Andrés Bello University (Santiago, Chile). Stock cultures of *Botrytis cinerea* were first inoculated in a Petri dish containing Potato Dextrose Agar (P.D.A., Difco) and incubated at 26 °C in darkness for 10 days. After incubation, Petri dishes were stored at 4 °C to be used as inoculum for every experiment.

To evaluate the antifungal effect exerted by **L1**, we determined the growth inhibition of mycelia. To this end, Petri dishes (90 mm) with a final volume of 15 mL of PDA (Difco) containing 0 (negative control), 2, 4, 6, 8 or 10 ppm of **L1** dissolved in DMSO (Merck) were used. As a positive control, fenhexamid (a commercial fungicide) was used at the same doses. Cultures were incubated for a total period of 23 days either at 4 ± 2 °C or 26 ± 2 °C. Periodical measures were performed to determine mycelial growth, which was recorded as growth diameter (cm) of the fungal colony. Both growth and measures were performed in darkness at 26 °C to prevent light-induced growth stimulation [93]. Every experiment was performed in biological triplicate.

To evaluate antimicrobial activity in bacteria (*Klebsiella pneumoniae*) and yeasts (*Rhodotorula* spp.), we determined the minimal inhibitory concentration, as previously described [8,9].

### 3.7. HeLa Cell Viability Assays

To determine cell viability, we performed an MTT assay, as previously described, using 25,000 cells/well in a 96-well plate [94]. Briefly, cells were cultured in Dulbecco’s Modified Eagle’s Medium (DMEM) containing 10% fetal bovine serum (FBS), 2 mM l-glutamine, 100 units/mL penicillin and 100 μg/mL streptomycin. Cells were maintained in 75 cm^2^ flasks in a 5% CO_2_-humidified atmosphere at 37 °C. The passage takes place every 2–3 days. All cell culture ingredients were purchased from Sigma-Aldrich. Toxicity of the respective complexes was determined using the 3-(4,5-dimethylthiazol-2-yl)-2,5-diphenyltetrazolium bromide (MTT) cell viability assay after 24 h of incubation with **L1**. MTT is a yellow compound that, when reduced by functioning mitochondria, produces purple formazan crystals that can be measured spectrophotometrically. For this purpose, MTT (Sigma-Aldrich) was dissolved in phosphate-buffered saline (PBS) to a concentration of 5 mg/mL and further diluted in culture medium (1:11). Cells were incubated with this MTT-solution for 3 h under standard culture conditions. Afterward, 155 μL of the solution was rejected, and 90 μL of DMSO was added. To completely dissolve the formazan salts plates were incubated for 10 min on a shaker and afterward quantified by measuring the absorbance at 535 nm with an ELISA microplate reader. Cell viability was calculated as the percentage of surviving cells compared to untreated control cells.

### 3.8. General Procedure of Synthesis of ***L1***

The (*E*)-2-{[(2-aminopyridin-2-yl)imino]-methyl}-4,6-di-*tert*-butyl-phenol (**L1**) compound was prepared by direct interaction between 1,2-diaminopyridine and 3,5-di-*tert*-butyl-2-ol-benzaldehyde (1:1) in methanol, at room temperature, according to a previously described non-template method [4,9]. *(E)-2-{[(2-aminopyridin-2-yl)imino]-methyl}-4,6-di-tert-butyl-phenol* (**L1**) yellow powder, 0.68 g (2.1 mmol, 75%); FTIR (ATR, cm^-1^): 3487 and 3255 (νOH), 2947 (νOH), 2947 and 2905 (νNH_2_), 2863 (νCH), 1607 (νHC=N), 1589 (νC=C). UV–Vis: (Hexane, room temperature) λ (nm)(ε) = 239 (25.63 × 10^3^ mol^-1^ dm^3^ cm^-1^), 277 (18.07 × 10^3^ mol^−1^ dm^3^ cm^-1^), 375 (15.26 × 10^3^ mol^−1^ dm^3^ cm^−1^); (Chloroform, room temperature) λ (nm)(ε)= 274 (32.78 × 10^3^ mol^−1^ dm^3^ cm^−1^), 372 (15.80 × 10^3^ mol^−1^ dm^3^ cm^−1^); (Methanol, room temperature) λ (nm)(ε)= 236 (23.77 × 10^3^ mol^−1^ dm^3^ cm^−1^), 274 (16.15 × 10^3^ mol^−1^ dm^3^ cm^−1^), 368 (13.69 × 10^3^ mol^−1^ dm^3^ cm^−1^); (DMSO, room temperature) λ (nm)(ε) = 378 (7.24 × 10^3^ mol^−1^ dm^3^ cm^−1^). ^1^H-NMR at 25 °C (400 MHz, acetone-_d6_): δ =1.35 [s; 9H;*tert*-Bu], 1.48 [s; 9H; *tert*-Bu], 5.51 [bs; 2H; -NH_2_], 6.71 [dd: *J* = 7.5; 5.0 Hz; 1H], 7.52–7.43 [m; 2H], 7.53 [s; 1H], 7.95 [dd: *J* = 4.9; 1H], 8.88 [s; 1H], 13.29 [s; -OH]; (400 MHz, CD_2_Cl_2_): δ =1.25 [s; 9H;*tert*-Bu], 1.40 [s; 9H; *tert*-Bu], 4.93 [bs; 2H; -NH_2_], 6.65 [dd: *J* = 7.6; 5.1 Hz; 1H], 7.31-712 [m; 2H], 7.41 [s; 1H], 7.87 [d: J=5.0; 1H], 8.57 [s; 1H], 12.94 [s; -OH]; (400 MHz, methanol-_d4_): δ =1.11 [s; 9H;*tert*-Bu], 1.24 [s; 9H; *tert*-Bu],6.60-6.40 [m; 1H], 7.18 [s; 2H], 7.28 [s; 1H], 7.24 [d: *J* = 6.7 Hz; 1H], 7.28 [s; 1H], 7.63 [d: *J* = 3.7 Hz; 1H], 8.57 [s; 1H]; ^1^H-NMR at 4 °C (400 MHz, CD_2_Cl_2_): δ = 1.25 [s; 9H;*tert*-Bu], 1.40 [s; 9H; *tert*-Bu], 4.89 [bs; 2H; -NH_2_], 6.70 [dd; 1H], 7.27–7.25 [m; 2H], 7.43 [d; 1H], 7.94 [d; 1H], 8.59 [s; 1H], 12.93 [s; -OH]; ^13^C-NMR at 25 °C (400 MHz, acetone-_d6_): δ = 28, 113, 119, 125, 127, 130, 136, 140, 146, 154, 158, 165; (400 MHz, CD_2_CL_2_): δ = 29, 30, 31, 34, 114, 118, 125, 127, 128, 130, 136, 141, 145, 153, 157, 165; (400 MHz, methanol-_d4_): δ = 28, 29, 30, 100, 113, 125, 127, 128, 144, 165. DEPT at 25 °C (400 MHz, acetone-_d6_): δ = 31, 113, 125, 127, 128, 146, 165; (400 MHz, CD_2_Cl_2_): δ = 29, 30, 31, 114, 125, 127, 128, 145, 165; (400 MHz, methanol-_d4_): δ = 28, 29, 30, 114, 125, 127, 128, 144, 165.

## 4. Conclusions

In this manuscript, we provided a further characterization of **L1**. We assessed the purity and corroborated the compound structure by a combination of techniques, including (^1^H and ^13^C-NMR, DEPT, HHCOSY, UV–vis and powder diffraction), and complemented with DFT and TD-DFT using standard CAM-B3LYP/TZ2P level of theory. Our analysis showed the high stability and strength of the intramolecular hydrogen bond in different solvents by performing noncovalent interactions index (NCI) studies. Furthermore, we determined the electrochemical profile of **L1** (and compared it with **L2**), providing a scan-rate study, demonstrating that the electrochemical calculated processes are controlled by mass transfer (diffusion) only for **L1**. These results show that small structural changes in these compounds could lead to changes in their properties. Finally, we showed that **L1** exerts an antifungal effect against two strains of *Botrytis cinerea* (mold), including a strain isolated directly from the field, primarily at 26 °C, remarking the potential of these king of Schiff bases as modulators of biological activities.

## Figures and Tables

**Table 1:** Crystal data for (*E*)-2-{[(2-aminopyridin-2-yl)imino]-methyl}-4,6-di-*tert*-butyl-phenol (**L1**) compound *.

Cell Constant	Values Powder Diffraction **
a (Å)	17.0520 (16.8457)
b (Å)	10.6445 (10.6227)
c (Å)	10.5946 (10.4817)
β	102.12 (101.268)
V (Å^3^)	1880.11 (1839.5)
Crystal Density (g cm^−3^)	1.150 (1.175)
R	7.73
R_wp_	20.44
R_expected_	2.64
R_bragg_	4.949

* Molecular weight (M): 325.45 (325.45). ** Values in parenthesis represent X-ray data reported [34]. Crystal system monoclinic *P21/c* (No 14), *Z* = 4, T = 298 K, radiation CuKa.

## Supplementary Materials

The following are available online, Figure S1: FTIR spectrum of L1, Figure S2: ^1^HNMR spectrum of L1 in acetone-_d6_ at 25 °C, Figure S3: Aromatic expanded of ^1^HNMR of L1 in acetone-_d6_ at 25 °C, Figure S4: ^13^CNMR spectrum of L1 in acetone-_d6_ at 25 °C, Figure S5: DEPT spectrum of L1 in acetone-_d6_ at 25 °C, Figure S6: HHCOSY spectrum of L1 in acetone-_d6_ at 25 °C, Figure S7: ^1^HNMR spectrum of L1 in CD_2_Cl_2_ at 25 °C, Figure S8: Aromatic expanded of ^1^HNMR of L1 in CD_2_Cl_2_ at 25 °C, Figure S9: ^13^CNMR spectrum of L1 in CD_2_Cl_2_ at 25 °C, Figure S10: DEPT spectrum of L1 in CD_2_Cl_2_ at 25 °C, Figure S11: HHCOSY spectrum of L1 in CD_2_Cl_2_ at 25 °C, Figure S12: ^1^HNMR spectrum of L1 in methanol-_d4_ at 25 °C, Figure S13: Aromatic expanded of ^1^HNMR of L1 in methanol-_d4_ at 25 °C, Figure S14: ^13^CNMR spectrum of L1 in methanol-_d4_ at 25 °C, Figure S15: DEPT spectrum of L1 in methanol-_d4_ at 25 °C, Figure S16: HHCOSY spectrum of L1 in methanol-_d4_ at 25 °C, Figure S17: Aromatic expanded ^1^HNMR spectrum of L1 in CD_2_Cl_2_ at 4 °C, Figure S18: Experimental UV-vis spectra in different organic solvents, Figure S19: Scan rate analysis for L1 electrochemical processes, Figure S20: Scan Rate analysis for L2 electrochemical processes, Figure S21: Calculated UV-vis absorption spectra for L1 in different solvents, Figure S22: Isosurface plots of the HOMO for L1, Figure S23: Isosurface plots of the LUMO for L1, Figure S24: Isosurface plots of the HOMO-2 for L1, Figure S25: MTT Assay in HeLa cells, Table S1: Positional (x, y, z) and displacement parameters for L1, Table S2: UV-Vis absorption spectra of L1 in different organic solvents, Table S3: Electrochemical signals description for pyridine Schiff bases of this study, Table S4: Scan Rate study results for determining diffusional control of described electrochemical processes (L1), Table S5: Scan Rate study results for determining diffusional control of described electrochemical processes (L2), Table S6: Most important transition energies calculated for L1 in different organic solvents, Table S7: Minimal inhibition concentration (µg/mL) of L1 (24 h of incubation). References [95,96,97,98,99,100,101,102,103] are cited in the Supplementary Materials.
