# Supplementary material for: Structural Characterization, DFT Calculation, NCI, Scan-Rate Analysis and Antifungal Activity against Botrytis cinerea of (E)-2-{[(2-Aminopyridin-2-yl)imino]-methyl}-4,6-di-tert-butylphenol (Pyridine Schiff Base)
Source: Molecules. 2020 Jun 13;25(12):2741. doi: 10.3390/molecules25122741 (PMC7357110; doi:10.3390/molecules25122741)
Supplement: Supplementary file 1 [file molecules-25-02741-s001.pdf]

molecules

Supplementary Materials

Structural Characterization, DFT Calculation, NCI, Scan-Rate Analysis and Antifungal Activity Against *Botrytis cinerea* of (*E*)-2-{[(2-Aminopyridin-2-yl)imino]-methyl}-4,6-di-*tert*-butylphenol (Pyridine Schiff Base)

Alexander Carreño [1,2,*], Dayán Páez-Hernández [1], Plinio Cantero-López [1], César Zúñiga [3,4], Jan Nevermann [5], Angélica Ramírez-Osorio [2], Manuel Gacitúa [6], Poldie Oyarzún [7], Felipe Sáez-Cortez [8], Rubén Polanco [8], Carolina Otero [9] and Juan A. Fuentes [5,*]

1 Center of Applied Nanosciences (CANS), Facultad de Ciencias Exactas, Universidad Andres Bello, República 330, 8370186, Santiago, Chile; alexander.carreno@unab.cl (A.C.); dayan.paez@unab.cl (D.P.-H.); pliniocantero@gmail.com (P.C.-L.)
2 FONDECYT de Inicio 11170637, Facultad de Ciencias Exactas, Universidad Andres Bello, República 330, 8370186, Santiago, Chile; alexander.carreno@unab.cl (A.C.); angelicamosorio@gmail.com (A.R.-O.)
3 Instituto de Ciencias Naturales, Facultad de Medicina Veterinaria y Agronomía, Universidad de Las Américas, Sede Providencia, Manuel Montt 948, 7500972, Santiago, Chile; cesar.zuniga.c@gmail.com
4 Facultad de Ciencias de la Salud, Universidad Central de Chile, Lord Cochrane 417, 8330507, Santiago, Chile; cesar.zuniga.c@gmail.com
5 Laboratorio de Genética y Patogénesis Bacteriana, Facultad de Ciencias de la Vida, Universidad Andres Bello, República 330, 8370186, Santiago, Chile; jnevermannschell@gmail.com (J.N.); jfuentes@unab.cl (J.A.F.)
6 Facultad de Química y Biología, USACH, Av. L.B. O'Higgins 3363, 7254758, Santiago, Chile; manuelgacitua@gmail.com
7 Laboratorio de Análisis de Sólido (LAS), Facultad de Ingeniería y Facultad de Ciencias Exactas, Universidad Andrés Bello, República 330, 8370186, Santiago, Chile; poldie.oyarzun@unab.cl
8 Centro de Biotecnología Vegetal (CBV), Laboratorio de Fitopatógenos Fúngicos, Facultad de Ciencias de la Vida, Universidad Andres Bello, República 330, 8370186, Santiago, Chile; fs.cortez92@gmail.com (F.S.-C.); rpolanco@unab.cl (R.P.)
9 Escuela de Química y Farmacia, Facultad de Medicina, Universidad Andres Bello, República 252, 8320000, Santiago, Chile; maria.otero@unab.cl
* Correspondence: alexander.carreno@unab.cl (A.C.); jfuentes@unab.cl (J.A.F.)

Supplementary Figures

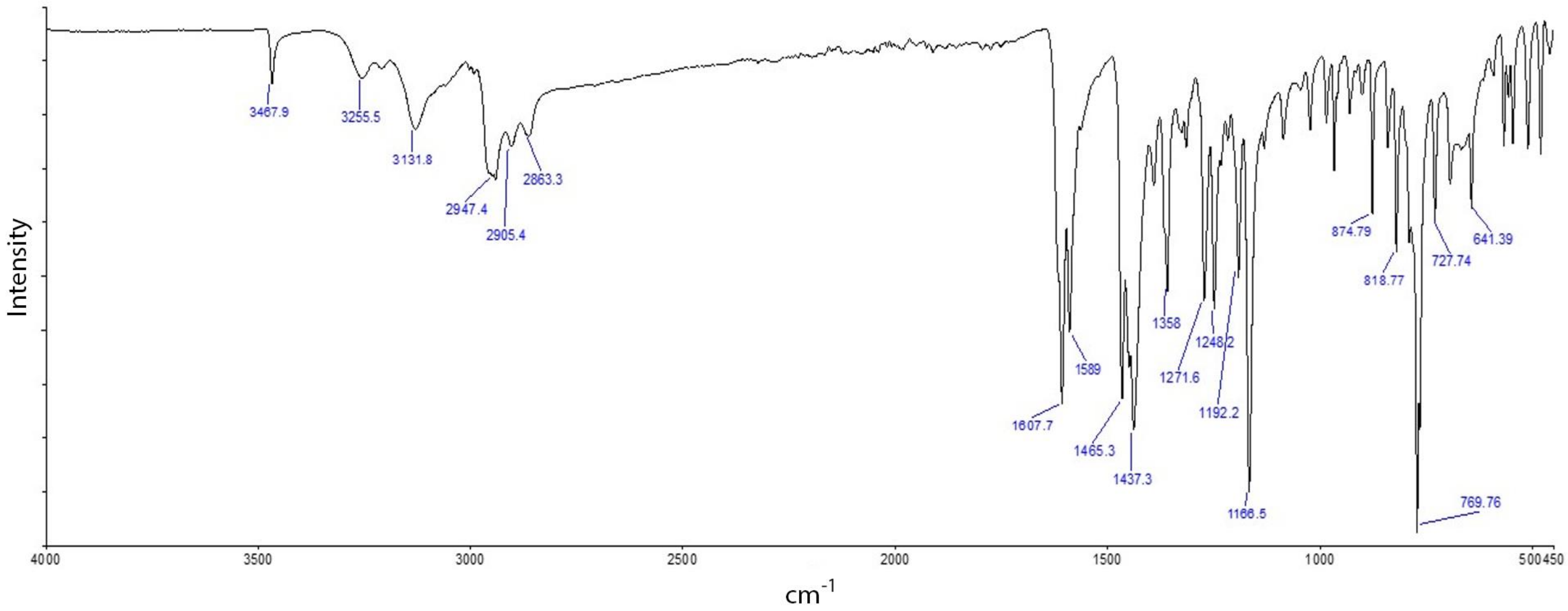

Figure S1. FTIR spectrum of **L1**.

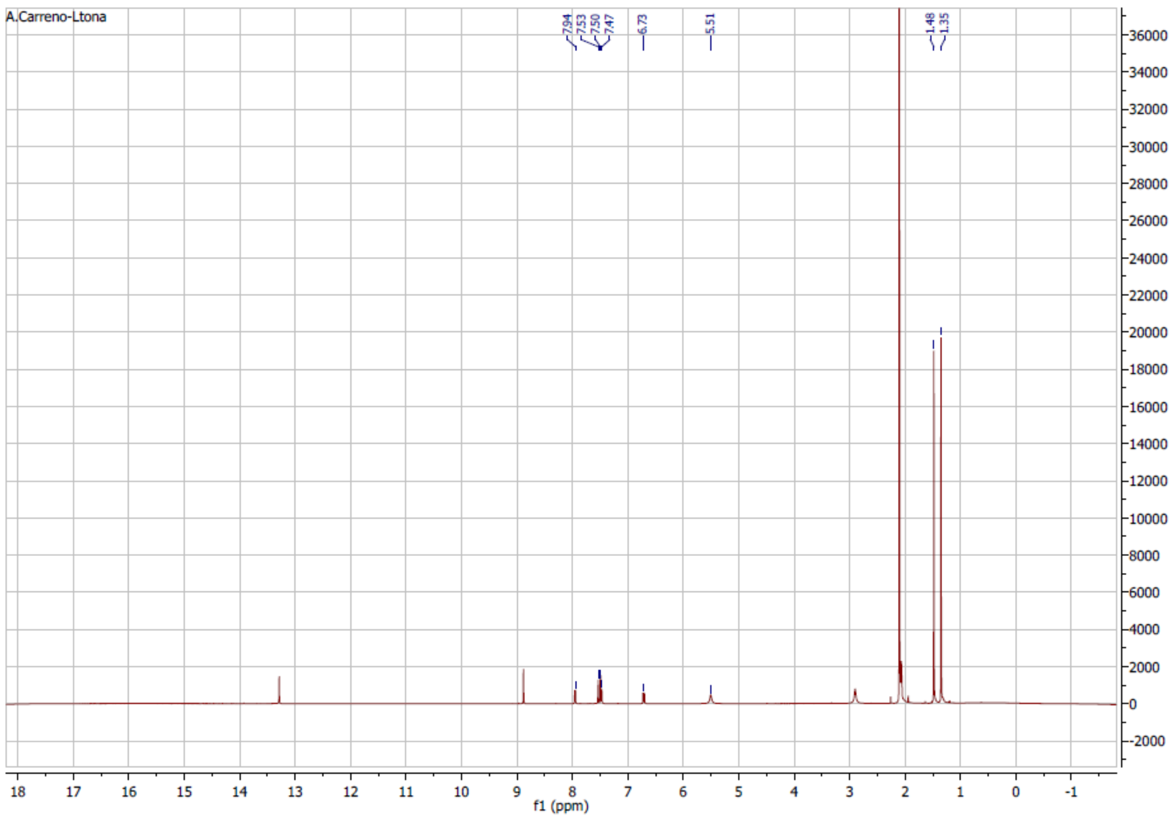

Figure S2. ^{1}HNMR spectrum of **L1** in acetone-d_6 at 25 °C.

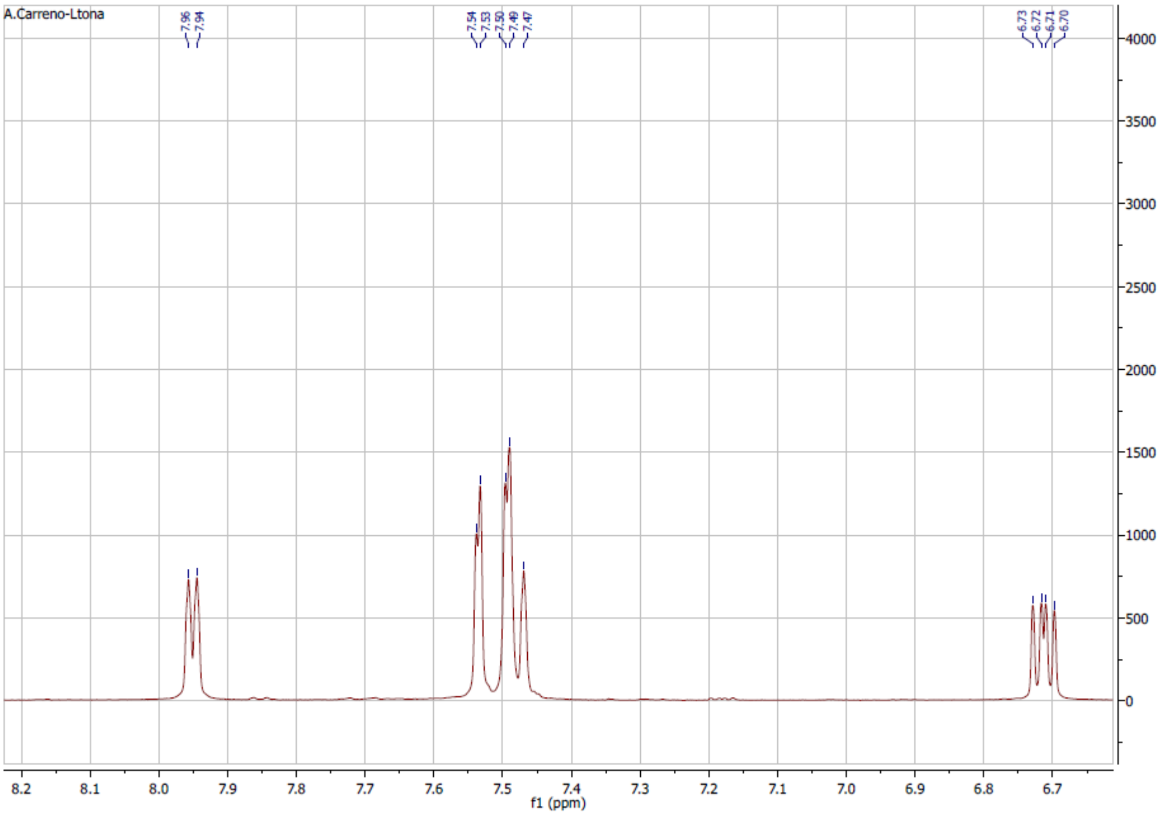

Figure S3. Aromatic expanded of ^{1}HNMR of **L1** in acetone-$_{d6}$ at 25 °C.

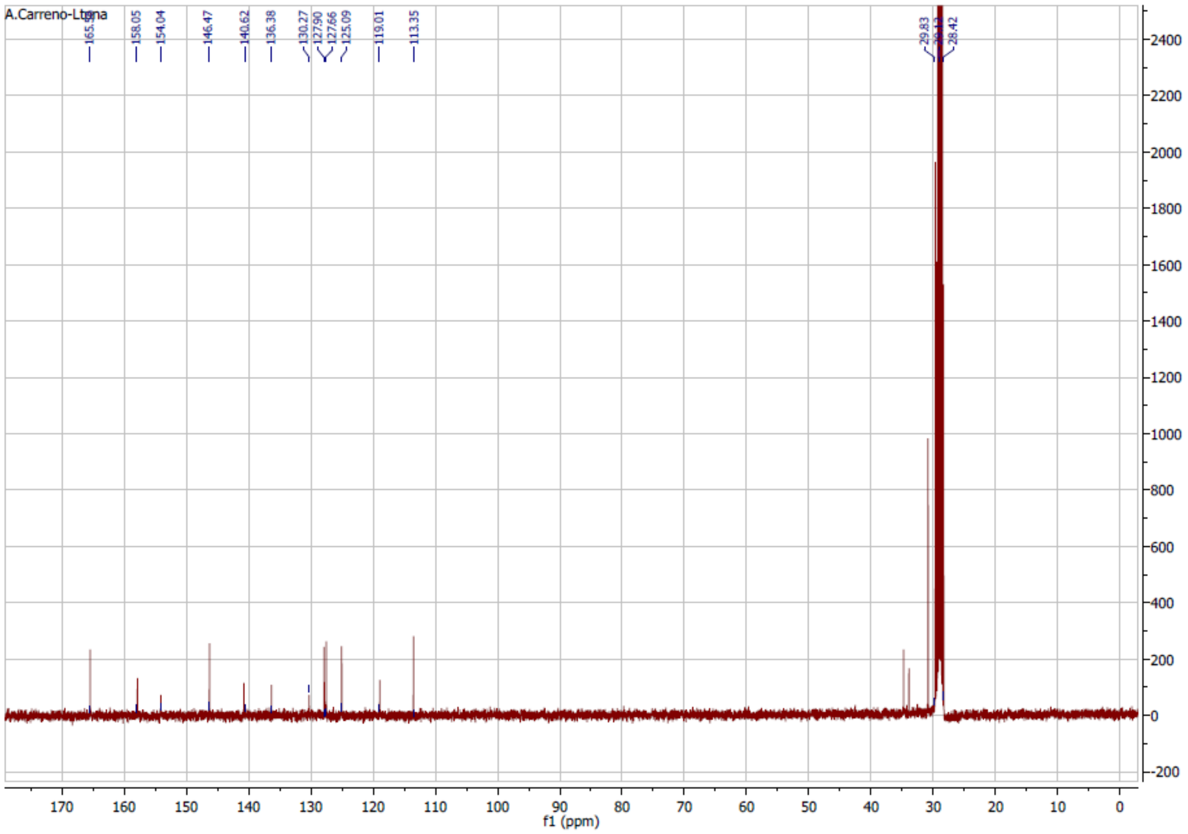

Figure S4. ^{13}CNMR spectrum of **L1** in acetone-$_{d6}$ at 25 °C.

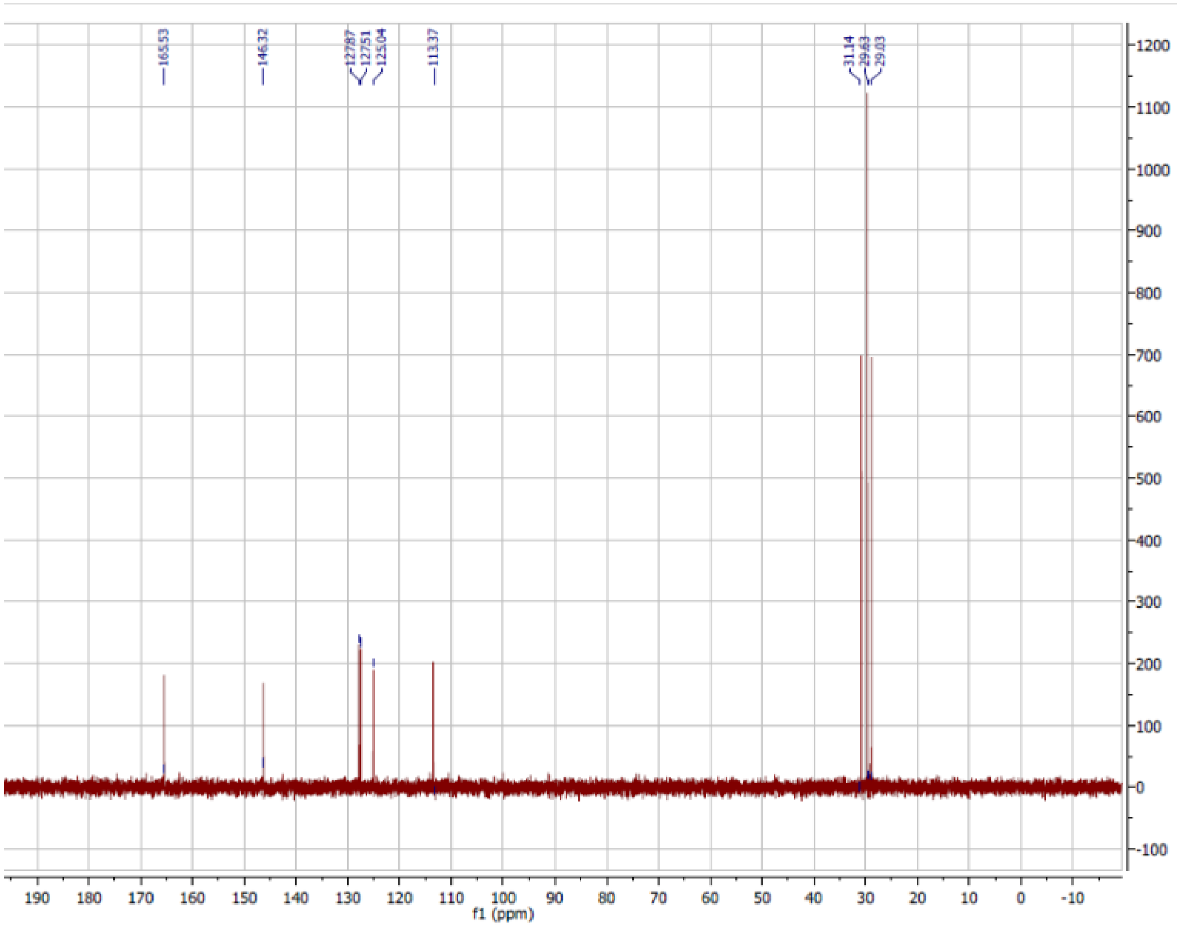

Figure S5. DEPT spectrum of **L1** in acetone-d6 at 25 °C.

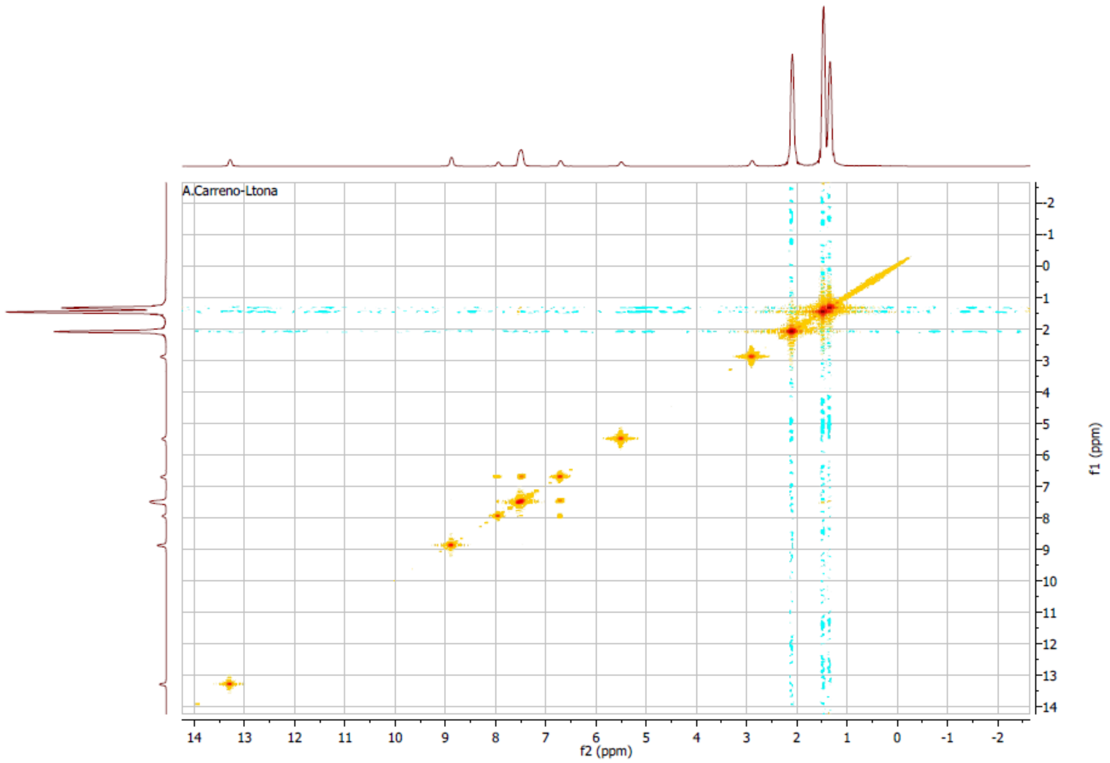

Figure S6. HHCOSY spectrum of **L1** in acetone-d_6 at 25 °C.

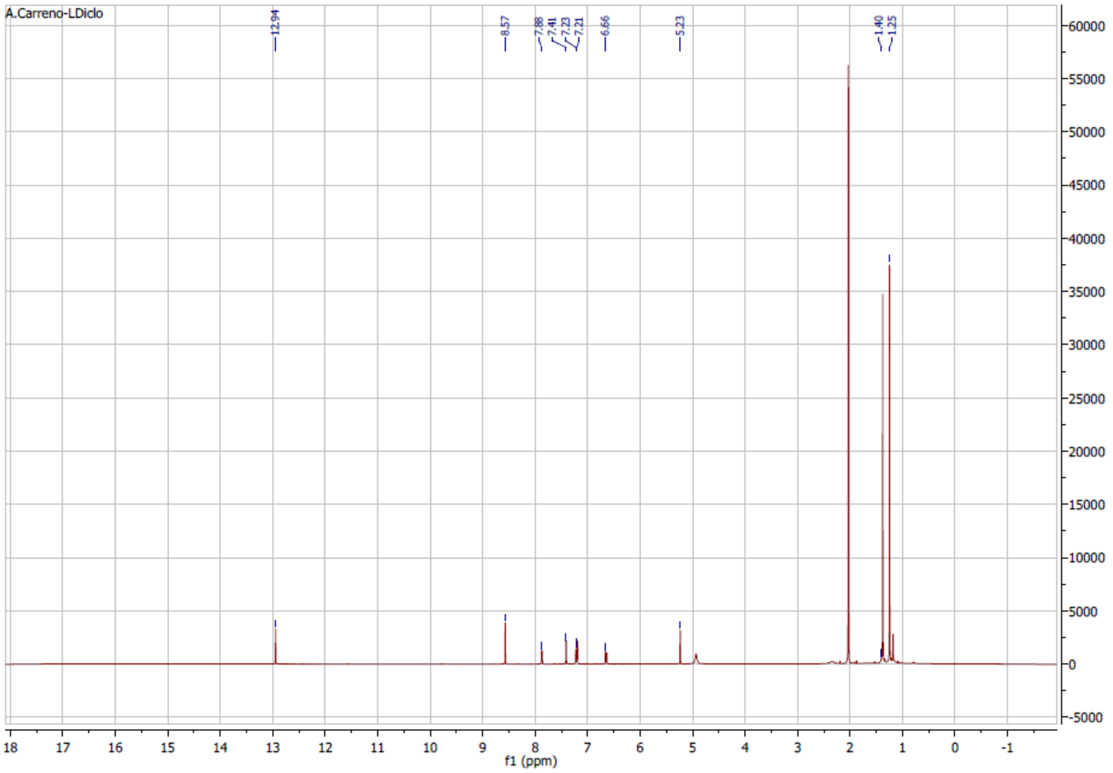

Figure S7. ^{1}HNMR spectrum of **L1** in CD_2Cl_2 at 25 °C.

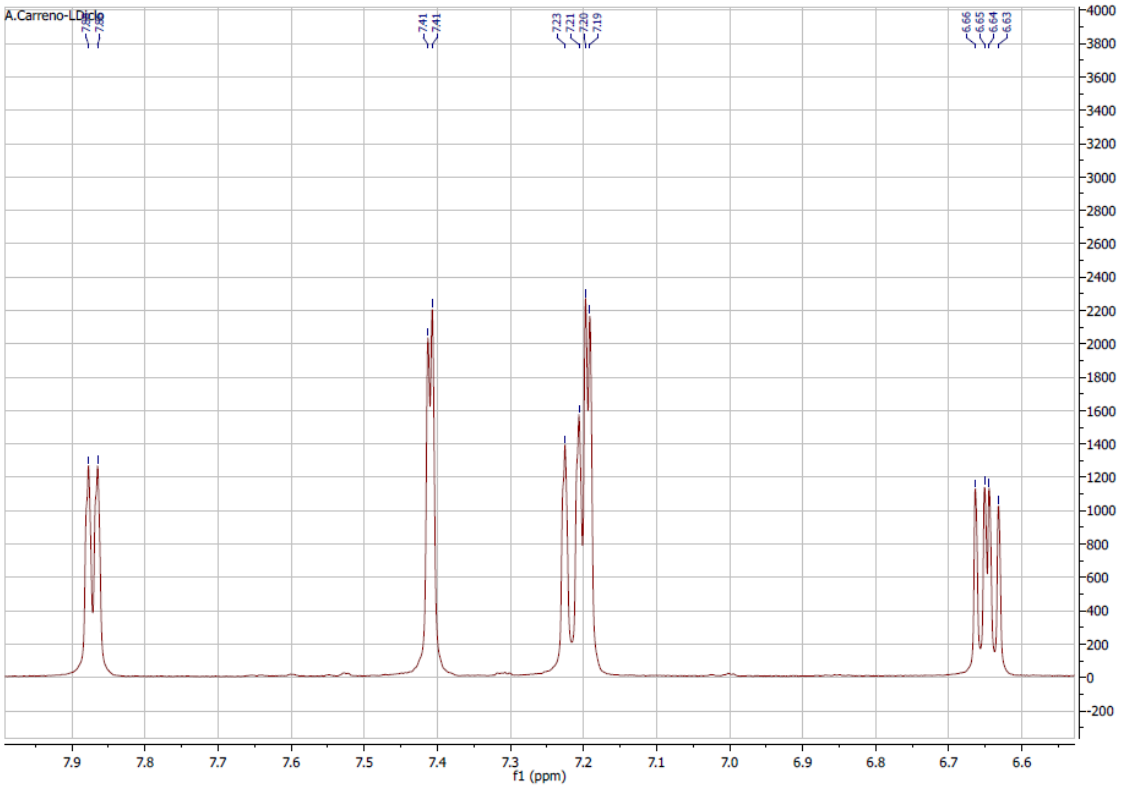

Figure S8. Aromatic expanded of ^{1}HNMR of **L1** in CD_2Cl_2 at 25 °C.

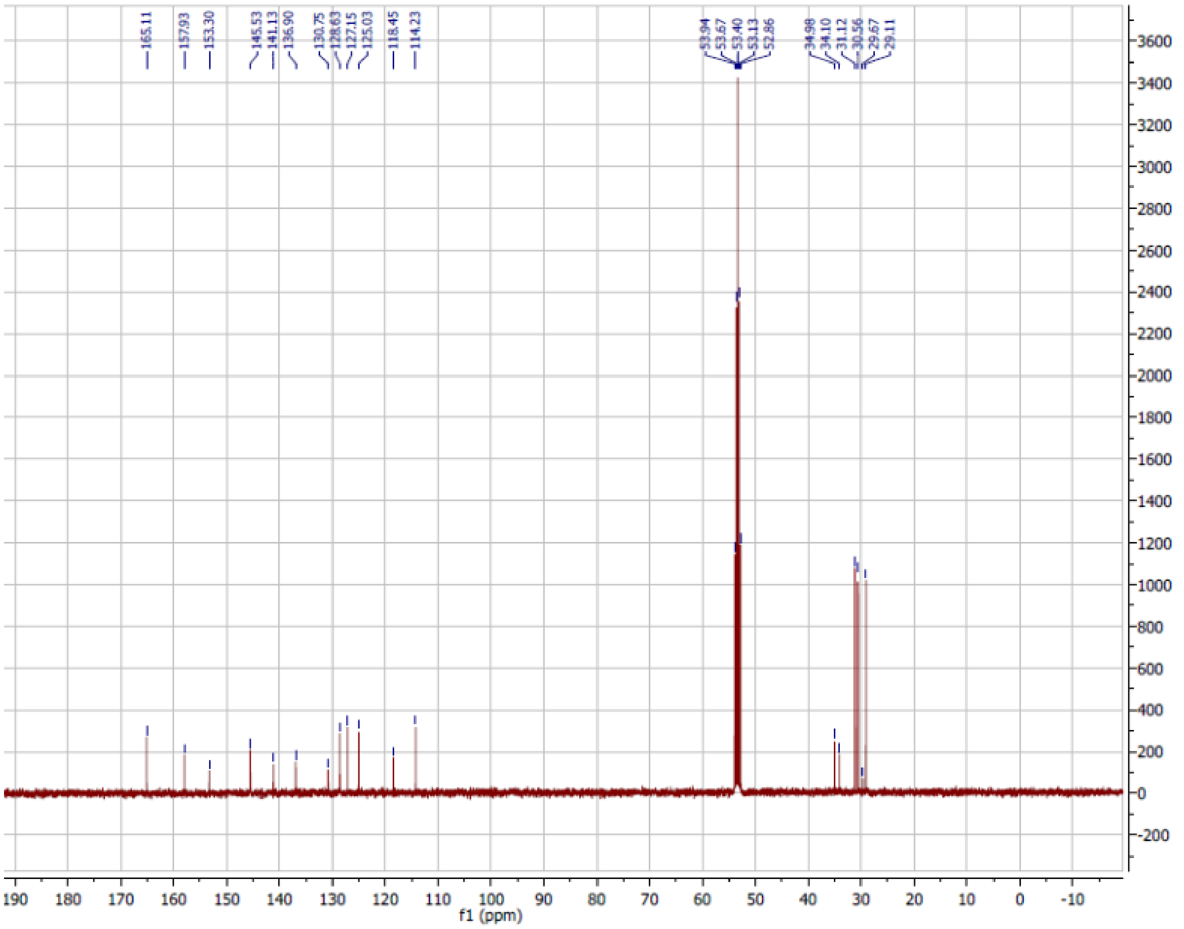

Figure S9. ^{13}CNMR spectrum of **L1** in CD_2Cl_2 at 25 °C.

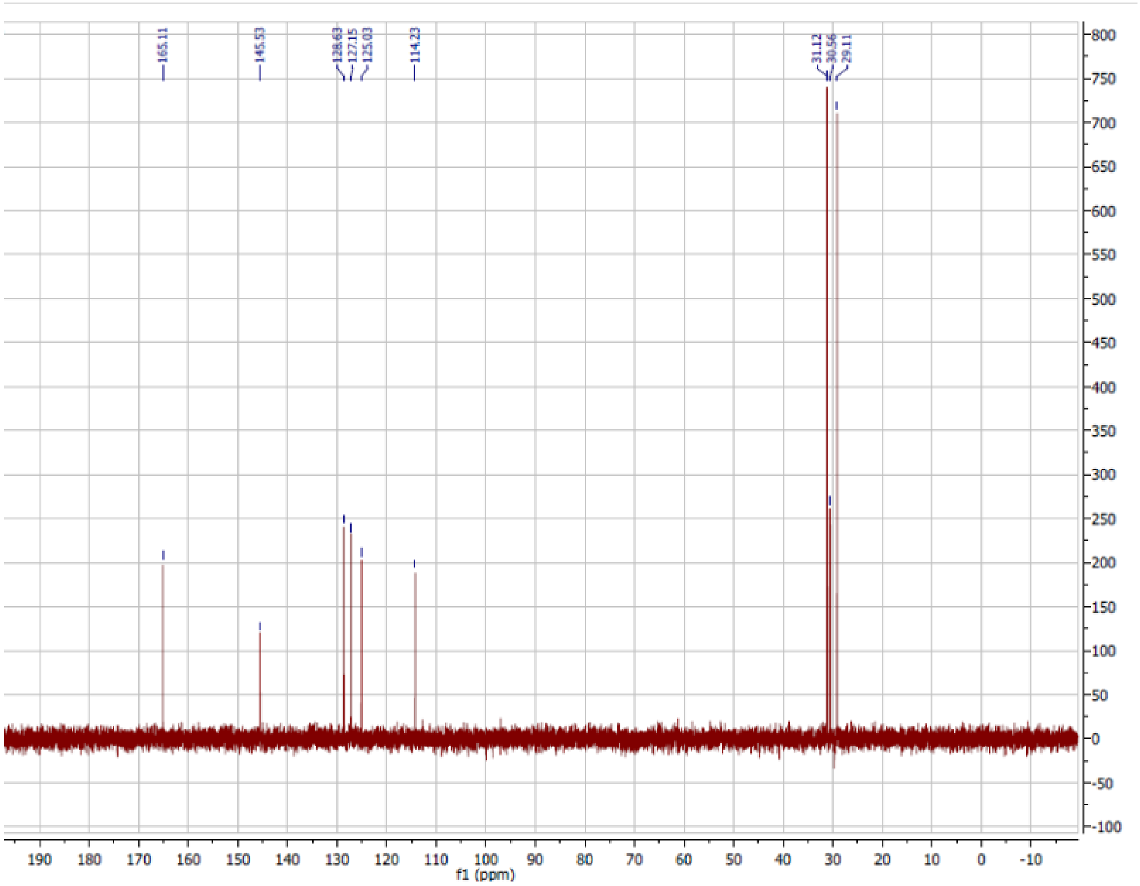

Figure S10. DEPT spectrum of **L1** in CD_2Cl_2 at 25 °C.

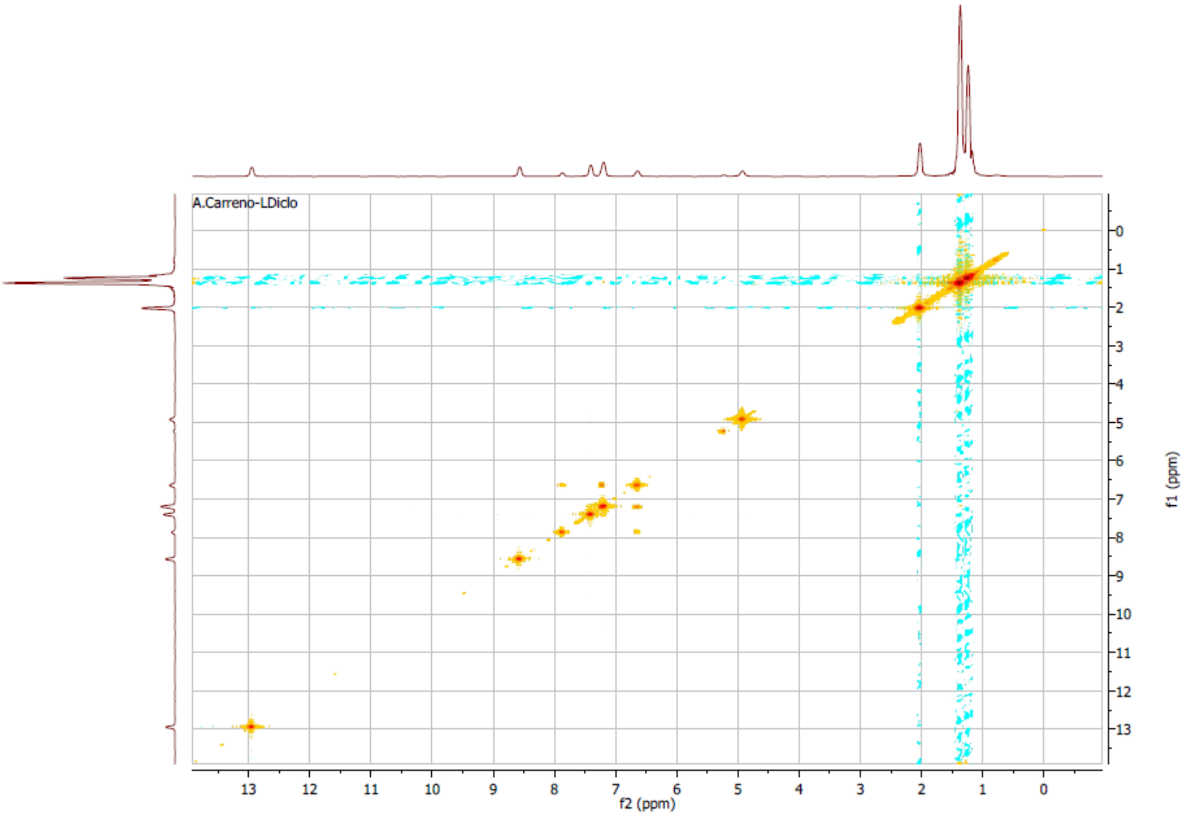

Figure S11. HHCOSY spectrum of **L1** in CD_2Cl_2 at 25 °C.

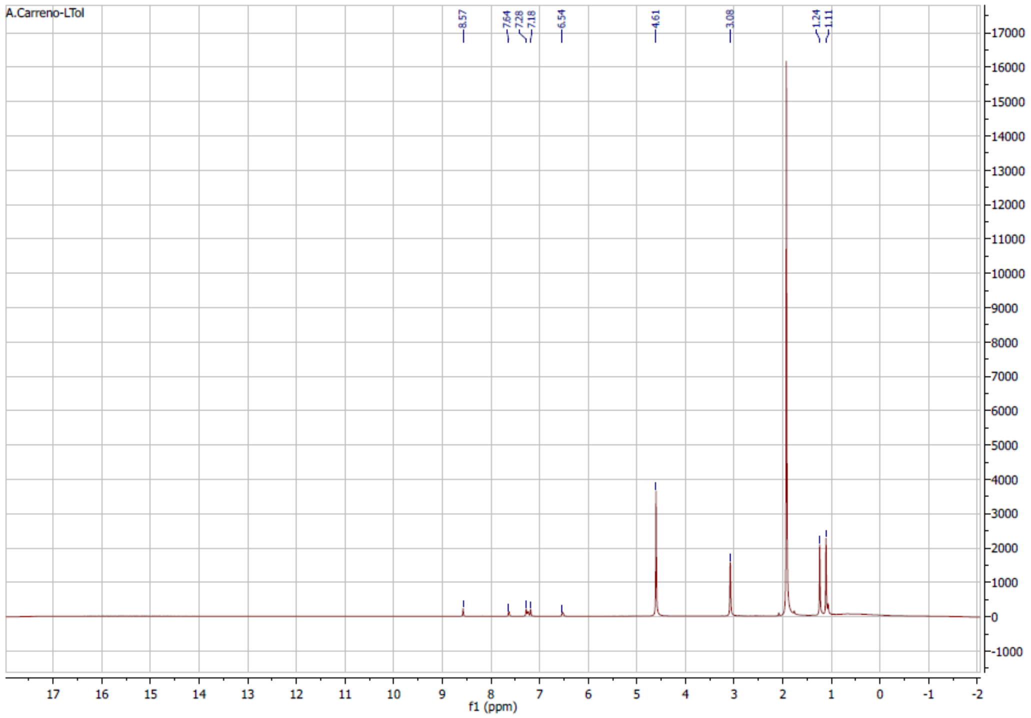

Figure S12. ^{1}HNMR spectrum of **L1** in methanol-$_{d4}$ at 25 °C.

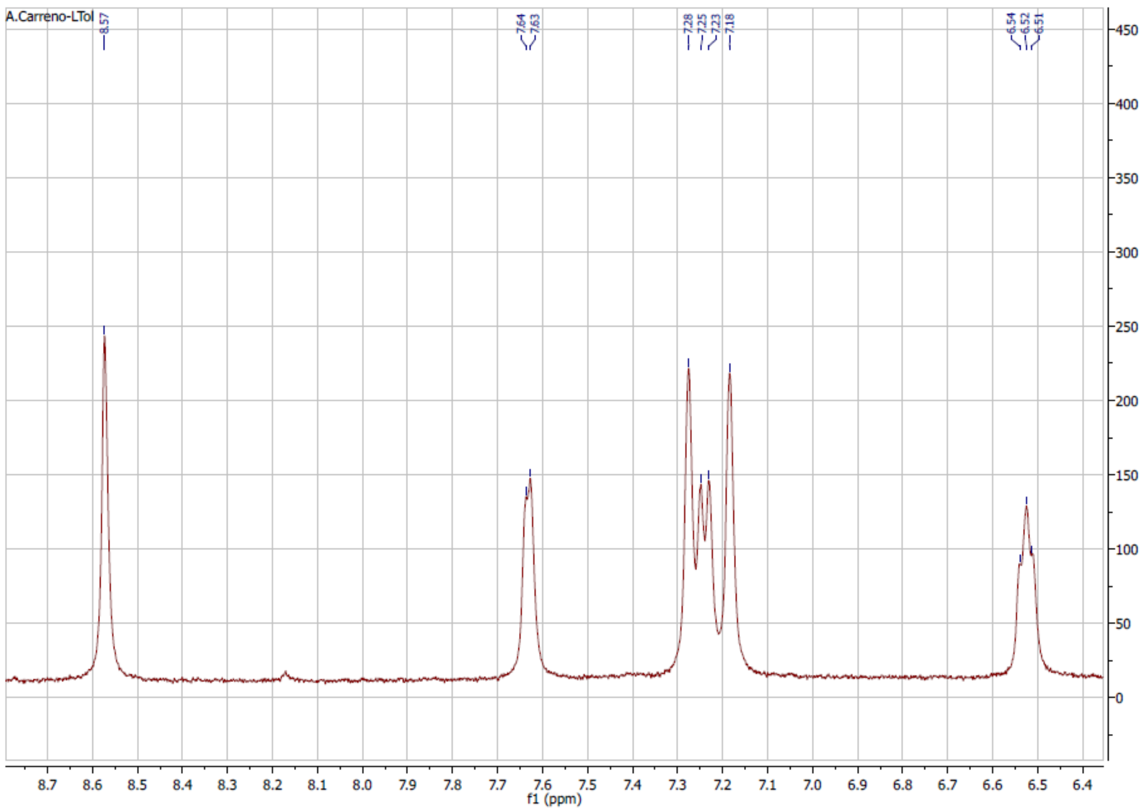

Figure S13. Aromatic expanded of ^{1}HNMR of **L1** in methanol-d4 at 25 °C.

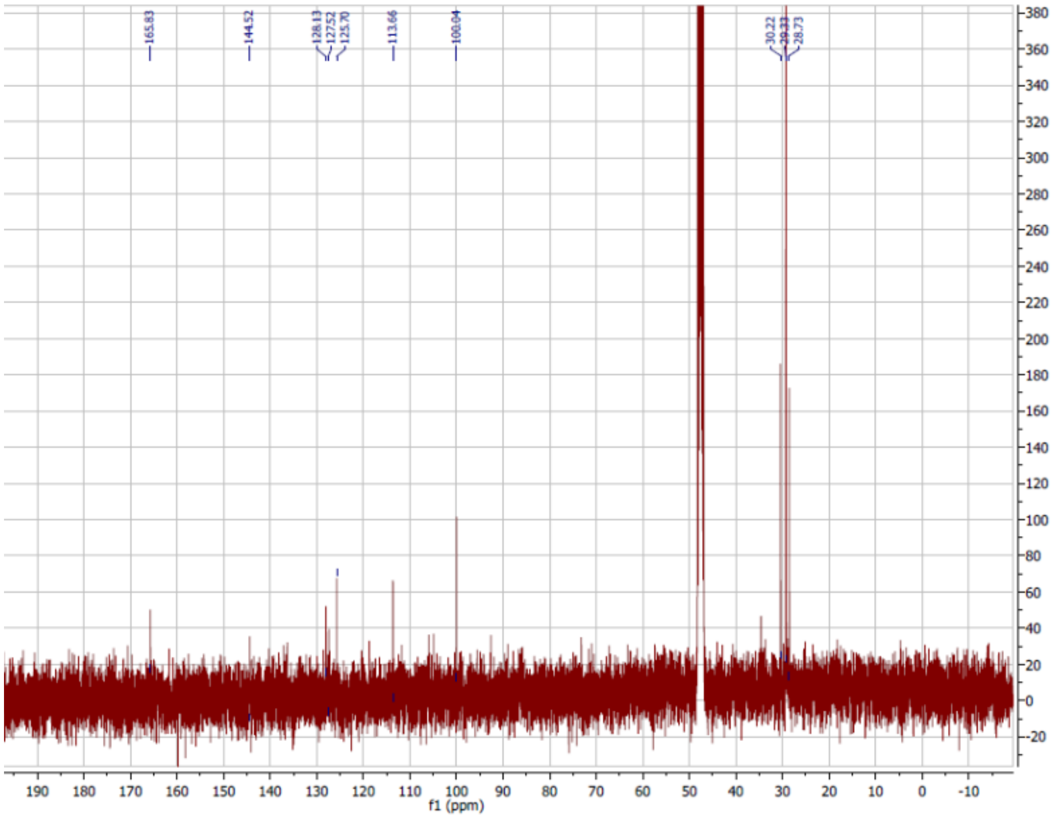

Figure S14. ^{13}CNMR spectrum of **L1** in methanol-$_{d4}$ at 25 °C.

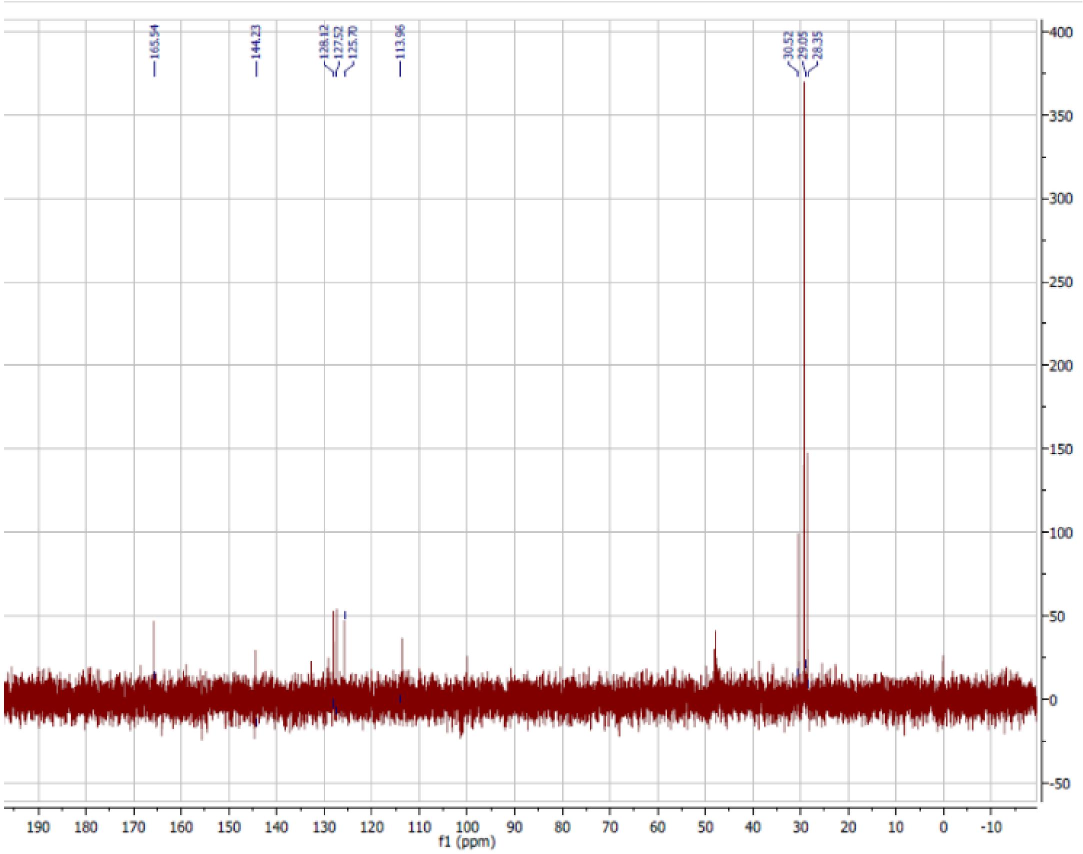

Figure S15. DEPT spectrum of **L1** in methanol-d4 at 25 °C.

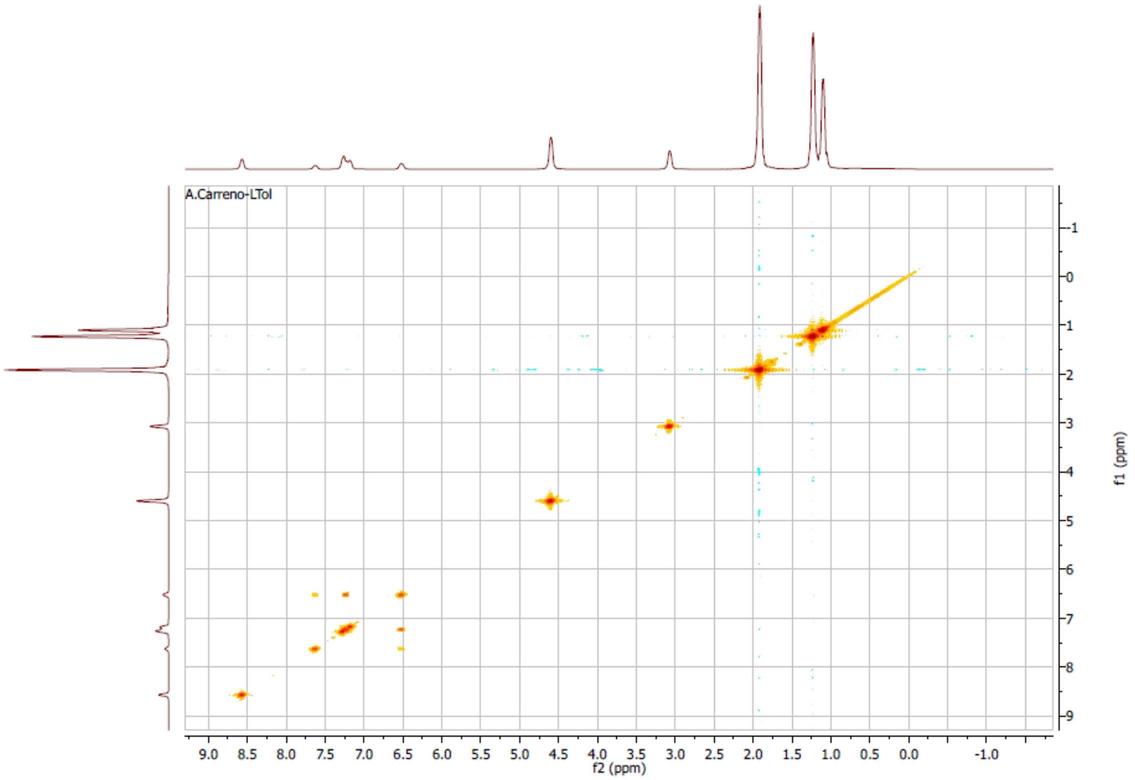

Figure S16. HHCOSY spectrum of **L1** in methanol-d4 at 25 °C.

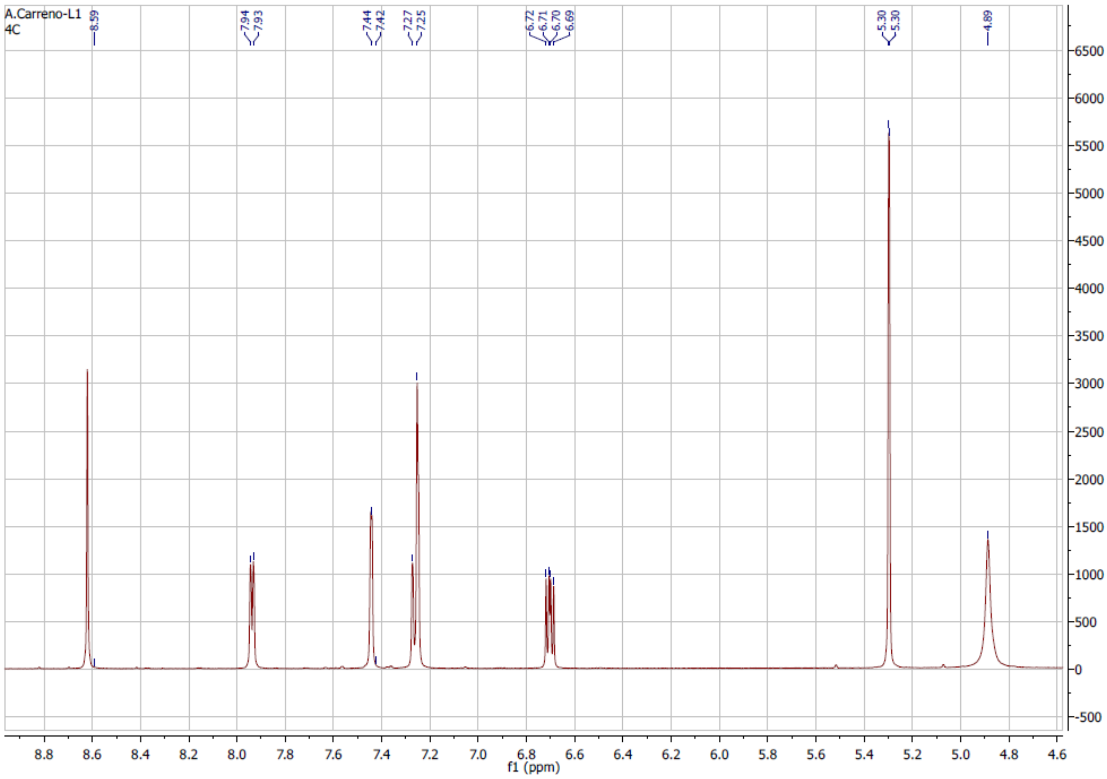

Figure S17. Aromatic expanded ^{1}HNMR spectrum of **L1** in CD_2Cl_2 at 4 °C.

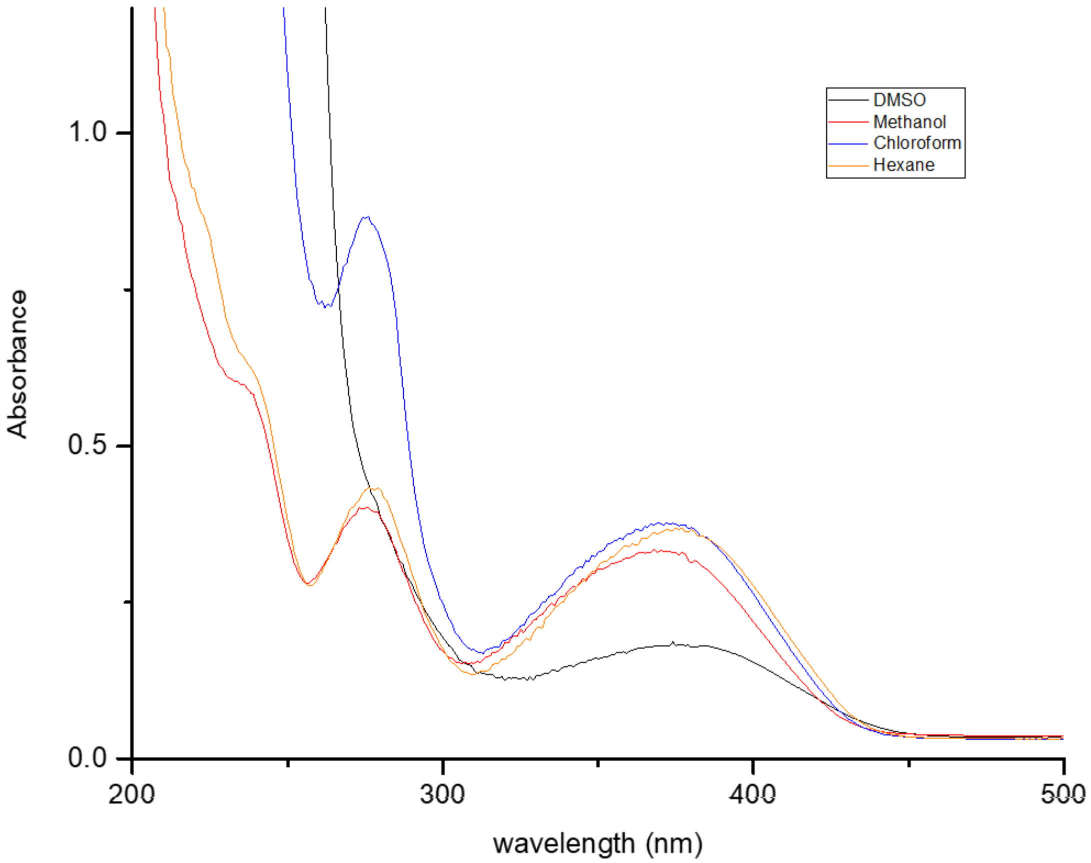

Figure S18. Experimental UV-vis spectra in different organic solvents, increasing polarity (hexane, chloroform, methanol, and DMSO) at room temperature.

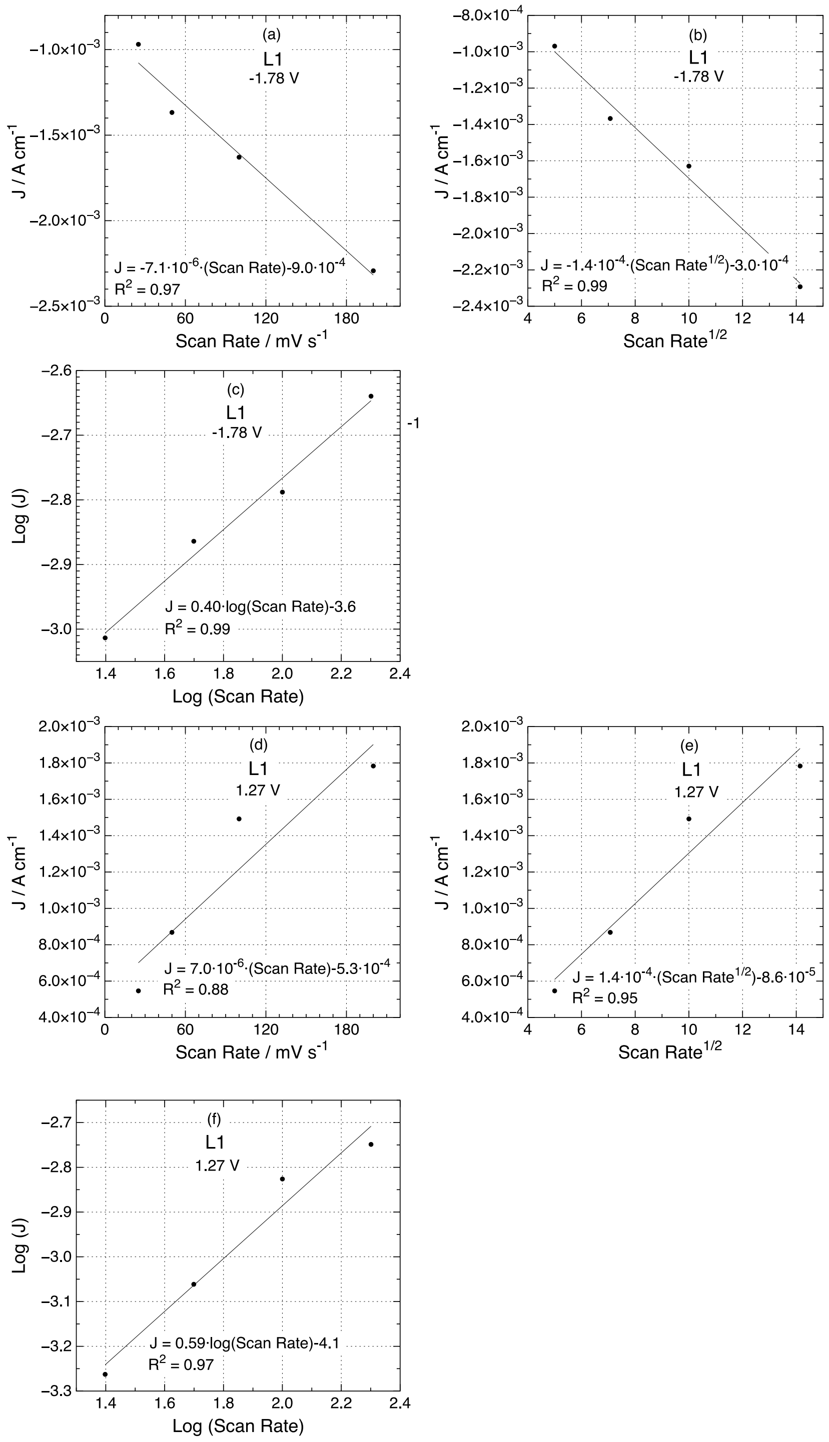

Figure S19. Scan rate analysis for **L1** electrochemical processes. Interphase: Same as Figure 3.

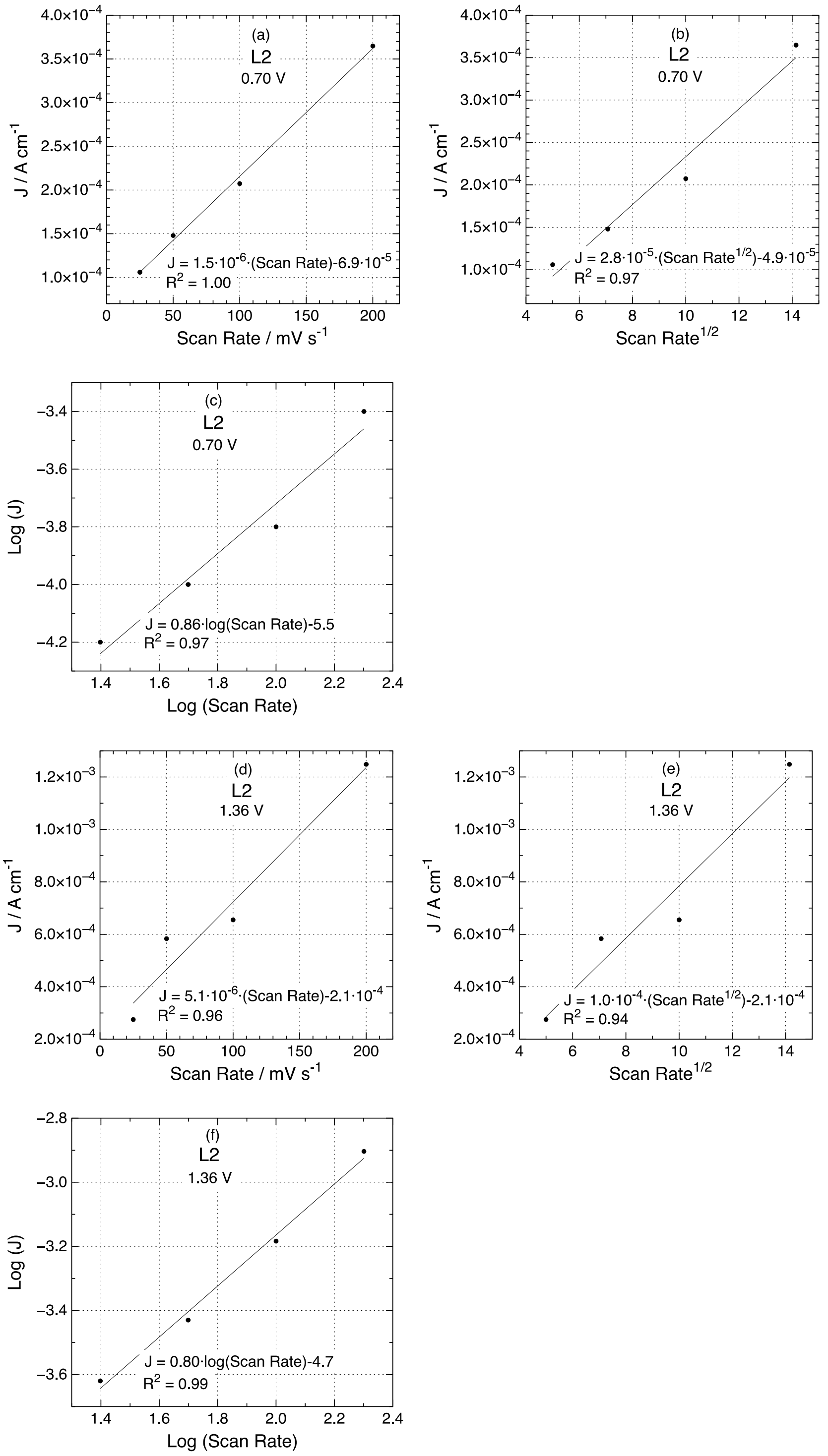

Figure S20. Scan Rate analysis for **L2** electrochemical processes. Interphase: Same as Figure 3.

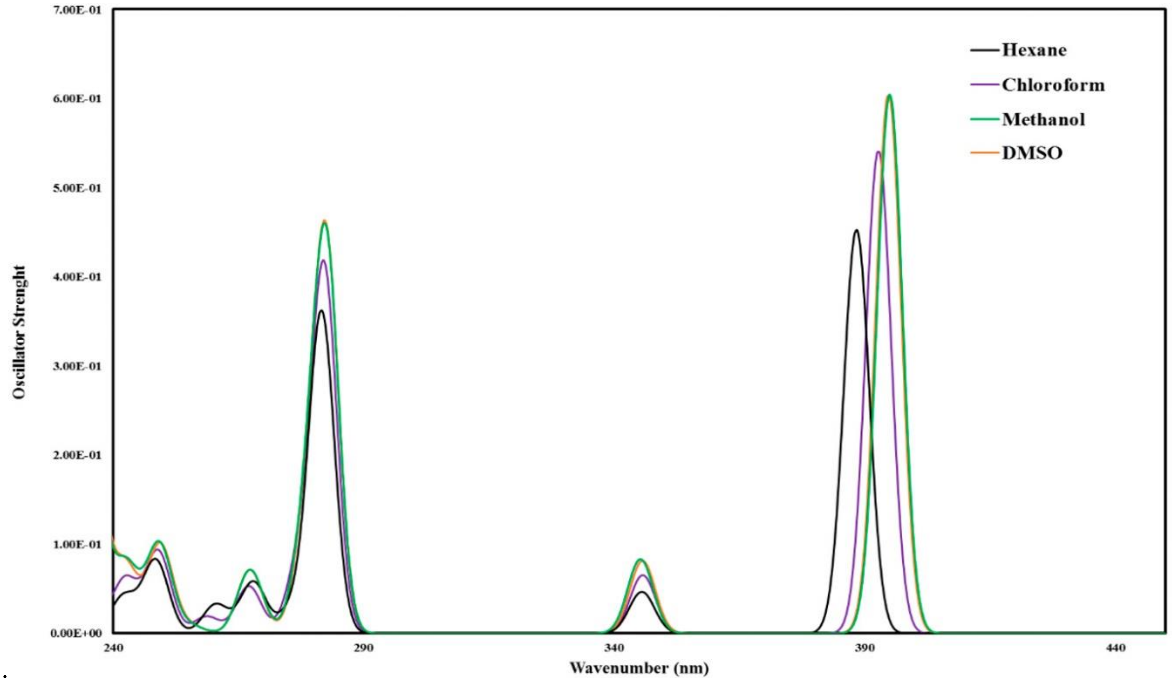

Figure S21. Calculated UV-vis absorption spectra for **L1** in different implicit solvents with increasing polarity: hexane, chloroform, methanol, and DMSO.

TD-DFT calculations were performed using the standard CAM-B3LYP/TZ2P level of theory. Solvation effects, simulated by the conductor-like screening model (COSMO) [1, 2].

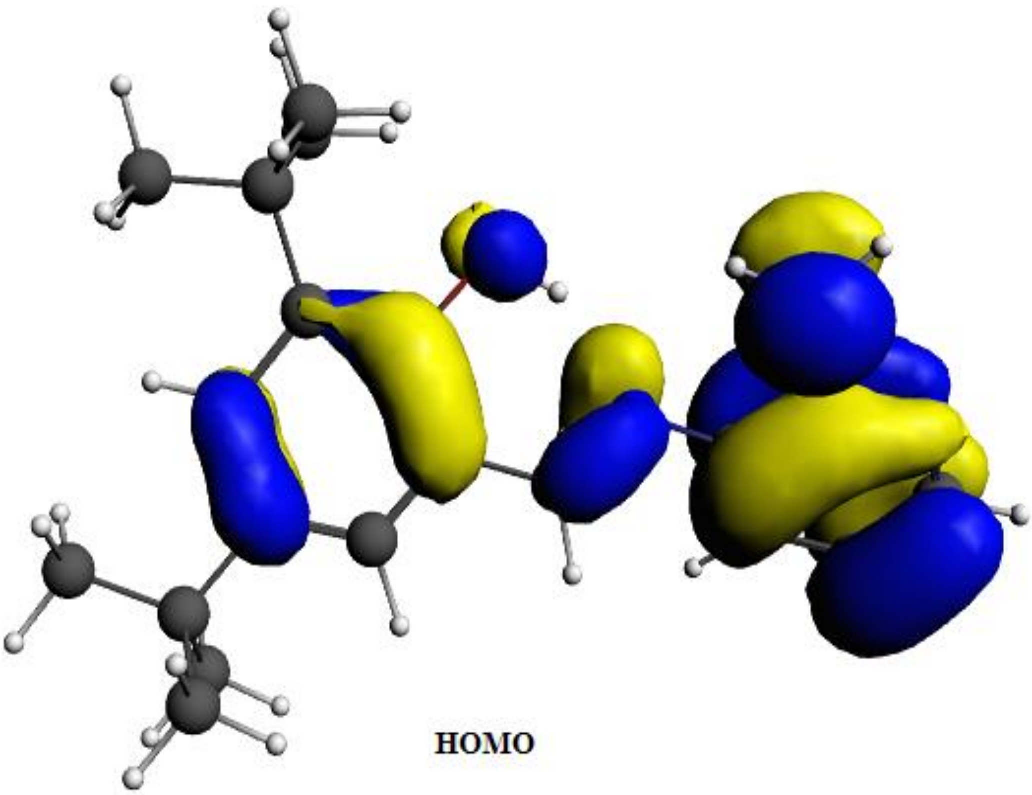

Figure S22. Isosurface plots of the HOMO for **L1.**

Theoretical computations were performed using density functional theory (DFT) with the B3LYP hybrid exchange/correlation (XC) functional and Gaussian basis set 6-311+G (2d,p) [3, 4].

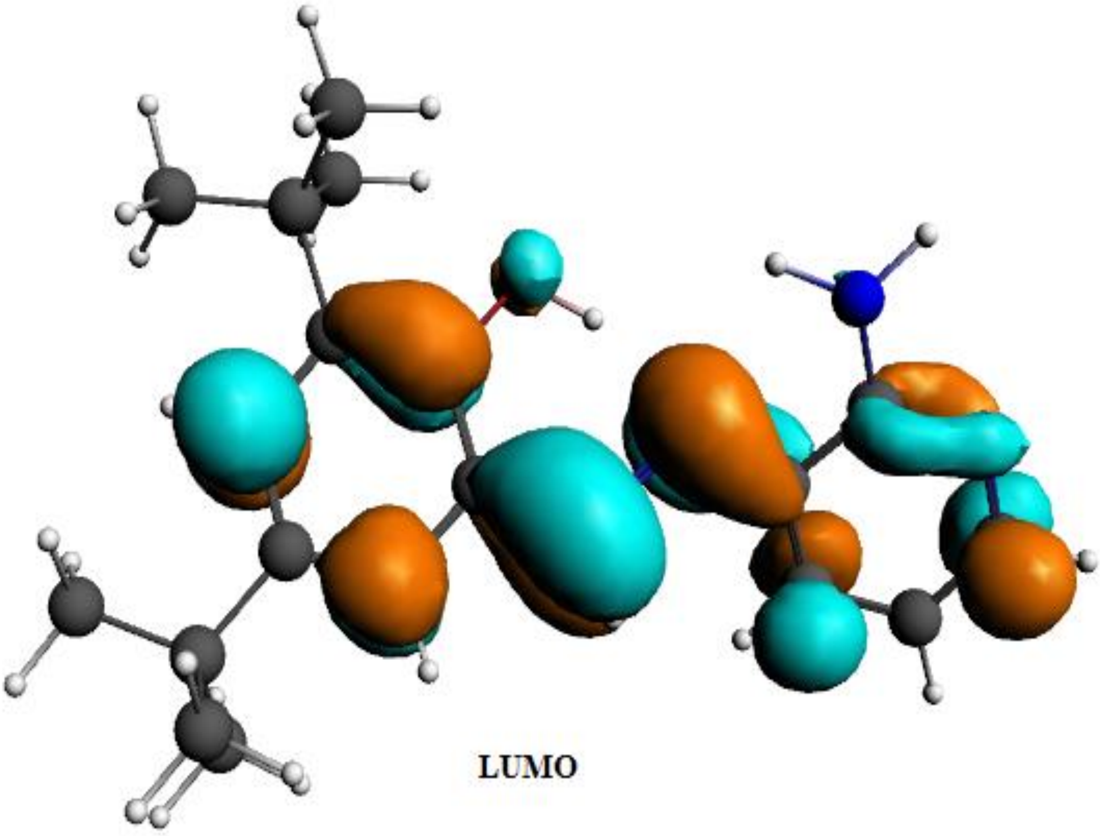

Figure S23. Isosurface plots of the LUMO for **L1.**

Theoretical computations were performed using density functional theory (DFT) with the B3LYP hybrid exchange/correlation (XC) functional and Gaussian basis set 6-311+G (2d,p) [3, 4].

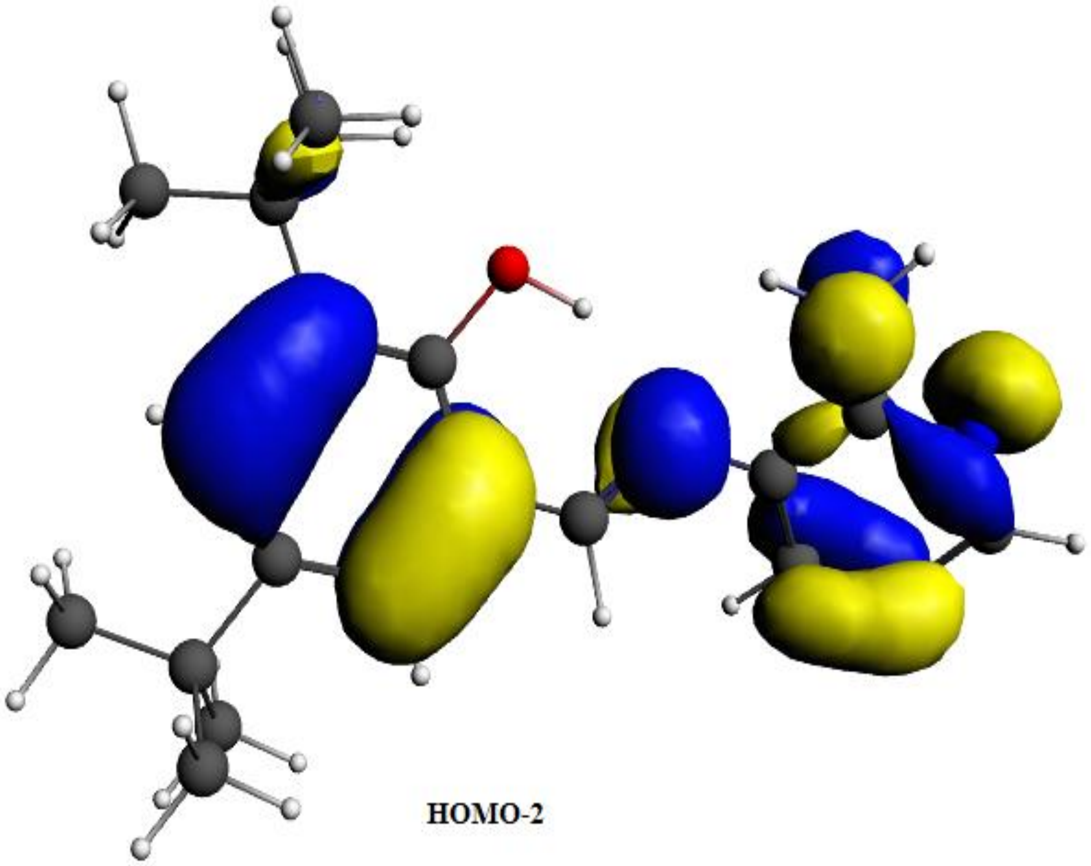

Figure S24. Isosurface plots of the HOMO-2 for **L1.**

Theoretical computations were performed using density functional theory (DFT) with the B3LYP hybrid exchange/correlation (XC) functional and Gaussian basis set 6-311+G (2d,p) [3, 4].

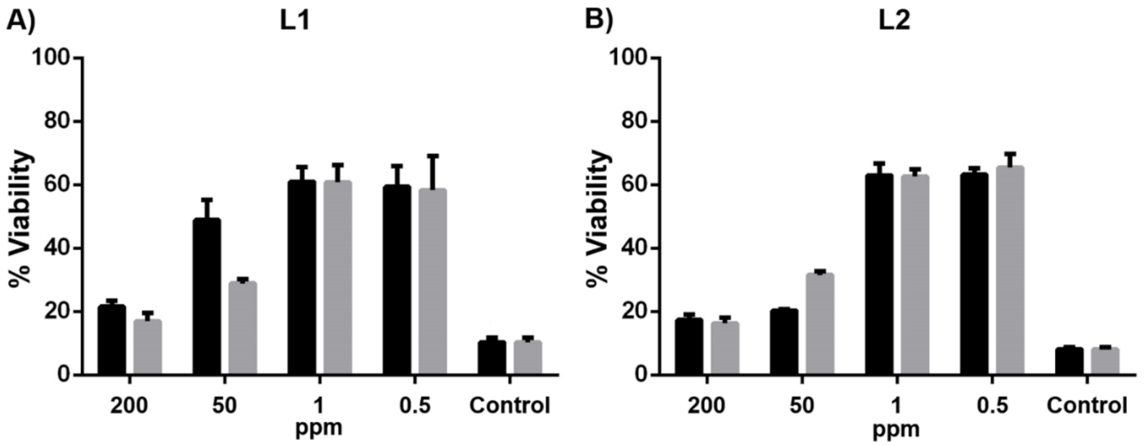

Figure S25. MTT Assay in HeLa cells. Cells were incubated 24 h with Schiff bases (A, **L1**; B, **L2**) before measuring cell viability. In all cases, compounds were tested at different concentrations (200 ppm + DMSO 50% v/v, 100 ppm + DMSO 25% v/v, 50 ppm + DMSO 12.5% v/v, or 25 ppm + DMSO 6.3% v/v) (black bars). The vehicle alone (DMSO) was also tested (50% v/v, 25% v/v, 12.5% v/v, or 6.3% v/v, respectively) (grey bars). The culture medium alone was used to set 100% viability. Control corresponded to DMSO 100% v/v. All these experiments were performed in biological triplicate.

For MTT assay, see [5, 6].

Supplementary Tables

Table S1. Positional (x, y, z) and displacement parameters for **L1**.

Atom	Np	x	y	z	Occ.	U iso/Beq.	Wyckoff symbol
C1	4	0.33545	0.84417	0.33556	1	2.029	4e
H1	4	0.37459	0.94429	0.24230	1	2.448	4e
C2	4	0.27364	0.96412	0.17400	1	1.966	4e
C3	4	0.21568	0.94016	0.16745	1	2.029	4e
C4	4	0.23234	0.81587	-0.03223	1	1.934	4e
C5	4	0.28514	0.68928	0.11164	1	2.1	4e
H5	4	0.37204	0.55128	0.06276	1	2.527	4e
C6	4	0.32395	0.75014	0.19495	1	1.998	4e
C7	4	0.27403	1.14626	0.37699	1	2.527	4e
C8	4	0.35160	1.14033	0.38834	1	3.079	4e
H8A	4	0.35507	1.06701	0.68542	1	4.579	4e
H8B	4	0.24883	1.12978	0.65241	1	4.579	4e
H8C	4	0.37472	0.9434	0.22987	1	4.579	4e
C9	4	0.20047	1.1425	0.29282	1	3.64	4e
H9A	4	0.11819	0.99795	0.22291	1	5.448	4e
H9B	4	0.13265	1.35418	0.19784	1	5.448	4e
H9C	4	0.07650	0.95712	0.15770	1	5.448	4e
C10	4	0.30881	1.12918	0.14100	1	3.774	4e
H10A	4	0.37469	0.94298	0.22511	1	5.685	4e
H10B	4	0.23293	1.24282	0.46667	1	5.685	4e
H10C	4	0.26412	1.3675	0.16078	1	5.685	4e
C11	4	0.42626	0.61177	0.28914	1	2.4	4e
C12	4	0.42088	0.64482	0.50998	1	3.782	4e
H12A	4	0.48387	0.60225	0.74619	1	5.685	4e
H12B	4	0.37573	0.55689	0.71548	1	5.685	4e
H12C	4	0.49382	0.6468	0.56858	1	5.685	4e
C13	4	0.35129	0.55479	0.34249	1	3.735	4e
H13A	4	0.37111	0.40791	0.26188	1	5.606	4e
H13B	4	0.28340	0.33962	0.38812	1	5.606	4e
H13C	4	0.35608	0.43213	0.18627	1	5.606	4e
C14	4	0.47336	0.64105	0.18316	1	3.822	4e
H14A	4	0.45257	0.6605	0.26729	1	5.764	4e

Atom	Np	x	y	z	Occ.	U iso/Beq.	Wyckoff symbol
H14B	4	0.45261	0.65627	0.26560	1	5.764	4e
H14C	4	0.45239	0.65962	0.25737	1	5.764	4e
C15	4	0.18988	0.67598	-0.03215	1	2.171	4e
H15	4	0.25215	0.64211	-0.06477	1	2.606	4e
C16	4	0.06221	0.88112	-0.31840	1	2.361	4e
C17	4	0.08584	0.75669	-0.24797	1	2.219	4e
C18	4	0.05118	0.66686	-0.19538	1	2.645	4e
H18	4	0.04209	0.59234	0.22390	1	3.158	4e
C19	4	-0.00149	0.60163	-0.34370	1	3.024	4e
H19	4	0.06203	0.64534	-0.52461	1	3.632	4e
C20	4	-0.03555	0.67756	-0.57434	1	3.135	4e
H20	4	0.07526	0.65881	-0.54724	1	3.79	4e
N1	4	0.13502	0.81102	-0.07618	1	2.282	4e
N2	4	0.00625	0.79541	-0.54461	1	2.803	4e
N3	4	0.09912	0.973	-0.36931	1	2.914	4e
H103	4	0.19755	0.93669	-0.25866	1	3.474	4e
H203	4	0.13583	0.88432	-0.54149	1	3.474	4e
O1	4	0.16787	1.00527	-0.01297	1	2.787	4e
H1A	4	0.16979	0.80368	-0.31899	1	4.185	4e

Crystal system monoclinic, *P21/c* (No 14), Z = 4, T = 298 K, radiation CuKa. For U iso/Beq. and Wyckoff symbol, see [7-9].

Table S2. UV-Vis absorption spectra of **L1** in different organic solvents with increased polarity at room temperature.

Solvent	Dielectric (at 20 ^oC)	Solubility in water (g/100 g)	λ / nm	ε × 10^3 (mol^{-1} dm^3 cm^{-1})	Transition nature
Hexane	1.89	0.0014	239<br>277<br>375	25.63<br>18.07<br>15.26	π → π*<br>π → π*<br>π → π* and n → π*
Chloroform	4.81	0.795	274<br>372	32.68<br>15.80	π → π*<br>π → π* and n → π*
Methanol	32.7	Miscible	236<br>274<br>368	23.77<br>16.15<br>13.69	π → π*<br>π → π*<br>π → π* and n → π*
DMSO	47.24	25.3	378	7.24	π → π* and n → π*

For Dielectric constant, see [10].

Table S3. Electrochemical signals description for pyridine Schiff bases of this study.

Compound	V vs. SCE			
	E_1^{Ox}	E_2^{Ox}	E_1^{Red}	E_2^{Red}
**L1**	+1.27$_{(irr-d)}$	—	-1.78$_{(irr.d)}$	—
**L2**	+0.70$_{(irr-nd)}$	+1.36$_{(irr-nd)}$	—	—

ox = oxidation. red = reduction. irr = irreversible. rev = reversible. d = diffusional. nd= not diffusional. For Electrochemical signals description see [11].

Table S4. Scan Rate study results for determining diffusional control of described electrochemical processes (**L1**).

		**-1.78** (irr)	**+1.27** (irr)
**Current-density peak vs Scan Rate**	m	-7.1×10^{-6}	7.0×10^{-6}
	n	-9.0×10^{-4}	-5.3×10^{-4}
	R^2	0.97	0.88
**Current-density peak vs (Scan Rate)^{½}**	m	-1.4×10^{-4}	1.4×10^{-4}
	n	-3.0×10^{-4}	-8.6×10^{-5}
	R^2	0.99	0.95
**Log(Current-density peak) vs Log(Scan Rate)**	m	0.40	0.59
	n	-3.6	-4.1
	R^2	0.99	0.97
**Diffusional or adsorption control?**		**Diffusional**	**Diffusional**

E_P corresponds to the oxidation (+) or reduction (-) peak potential value defined by the working-window study.

"irr" and "rev" stand for reversible or irreversible electrochemical process, respectively.

"m", "n" and "R^2" are the slope, intercept and linear regression coefficient, respectively.

.

Table S5. Scan Rate study results for determining diffusional control of described electrochemical processes (**L2**).

E_p / V vs SCE		+0.70 (irr)	+1.36 (irr)
Scan-rate vs current-density peak	m	1.5×10^{-6}	5.1×10^{-6}
	n	-6.9×10^{-5}	-2.1×10^{-4}
	R^2	1.00	0.96
(Scan-rate)^{½} vs current-density peak	m	2.8×10^{-5}	1.0×10^{-4}
	n	-4.9×10^{-5}	-2.1×10^{-4}
	R^2	0.97	0.94
Log(Current-density peak) vs Log(Scan Rate)	m	0.86	0.80
	n	-5.5	-4.7
	R^2	0.97	0.99
**Diffusional or adsorption control?**		**Adsorption**	**Adsorption**

E_P corresponds to the oxidation (+) or reduction (-) peak potential value defined by the working-window study.

"irr" and "rev" stand for reversible or irreversible electrochemical process, respectively.

"m", "n" and "R^2" are the slope, intercept and linear regression coefficient, respectively.

Table S6. Most important transition energies calculated for **L1** in different organic solvents, listed according to increasing polarity.

Solvent	λ_{exp} (nm)	λ_{calc} (nm)	Transition	f	Assignment
**Hexane**	239<br>277	281	HOMO-2 → LUMO (88%)	0.360	π → π*
	375	388	HOMO → LUMO (98%)	0.450	π → π* and<br>n → π*
**Chloroform**	274	282	HOMO-2 → LUMO (92%)	0.408	π → π*
	372	392	HOMO → LUMO (99%)	0.542	π → π* and<br>n → π*
**Methanol**	236<br>274	282	HOMO-2 → LUMO (94%)	0.440	π → π*
	368	395	HOMO → LUMO (99%)	0.604	π → π* and<br>n → π*
**DMSO**	---	283	HOMO-2 → LUMO (93%)	0.430	π → π*
	378	401	HOMO → LUMO (98%)	0.605	π → π* and<br>n → π*

TD-DFT calculations were performed using the standard CAM-B3LYP/TZ2P level of theory. Solvation effects were simulated by the conductor-like screening model (COSMO).

Table S7. Minimal inhibition concentration (μg/ml) of **L1** (24 h of incubation).

	**Bacteria (*K. pneumoniae*)**	**Yeast (*Rhodotorula* spp.)**
**L1**	-	-
Fluconazole*	ND	5.8
Chloramphenicol**	6.25	ND

- The inhibition was indistinguishable from de vehicle (DMSO) alone. * Antifungal agent (azole, control). ** Antibacterial agent (antibiotic, control).

References

1. Yanai, T.; Tew, D. P.; Handy, N. C., A new hybrid exchange–correlation functional using the Coulomb-attenuating method (CAM-B3LYP). *Chemical Physics Letters* **2004,** 393, (1-3), 51-57.
2. Klaumunzer, B.; Kroner, D.; Saalfrank, P., (TD-)DFT calculation of vibrational and vibronic spectra of riboflavin in solution. *J Phys Chem B* **2010,** 114, (33), 10826-34.
3. Bircher, M. P.; Rothlisberger, U., Plane-Wave Implementation and Performance of a-la-Carte Coulomb-Attenuated Exchange-Correlation Functionals for Predicting Optical Excitation Energies in Some Notorious Cases. *J Chem Theory Comput* **2018,** 14, (6), 3184-3195.
4. Plumley, J. A.; Dannenberg, J. J., A comparison of the behavior of functional/basis set combinations for hydrogen-bonding in the water dimer with emphasis on basis set superposition error. *J Comput Chem* **2011,** 32, (8), 1519-27.
5. Yoon, J. M.; Koppula, S.; Huh, S. J.; Hur, S. J.; Kim, C. G., Low concentrations of doxycycline attenuates FasL-induced apoptosis in HeLa cells. *Biological research* **2015,** 48, 38.
6. Schinkovitz, A.; Kaur, A.; Urban, E.; Zehl, M.; Pachnikova, G.; Wang, Y.; Kretschmer, N.; Slaninova, I.; Pauli, G. F.; Franzblau, S. G.; Krupitza, G.; Bauer, R.; Kopp, B., Cytotoxic constituents from Lobaria scrobiculata and a comparison of two bioassays for their evaluation. *J Nat Prod* **2014,** 77, (4), 1069-73.
7. Ramkumar, T.; Selvakumar, M.; Vasanthsankar, R.; Sathishkumar, A. S.; Narayanasamy, P.; Girija, G., Rietveld refinement of powder X-ray diffraction, microstructural and mechanical studies of magnesium matrix composites processed by high energy ball milling. *Journal of Magnesium and Alloys* **2018,** 6, (4), 390-398.
8. Nespolo, M.; Aroyo, M. I.; Souvignier, B., Crystallographic shelves: space-group hierarchy explained. *J Appl Crystallogr* **2018,** 51, (5), 1481-1491.
9. Janner, A.; Janssen, T.; de Wolff, P. M., Wyckoff positions used for the classification of Bravais classes of modulated crystals. *Acta Crystallographica Section A Foundations of Crystallography* **1983,** 39, (5), 667-670.
10. Mohsen-Nia, M.; Amiri, H.; Jazi, B., Dielectric Constants of Water, Methanol, Ethanol, Butanol and Acetone: Measurement and Computational Study. *Journal of Solution Chemistry* **2010,** 39, (5), 701-708.
11. Lai, B.-C.; Wu, J.-G.; Luo, S.-C., Revisiting Background Signals and the Electrochemical Windows of Au, Pt, and GC Electrodes in Biological Buffers. *ACS Applied Energy Materials* **2019,** 2, (9), 6808-6816.
